# Adaptive spectral–thermal illumination management for protected tomato cultivation: a fused deep learning and pareto-based decision framework

**DOI:** 10.3389/fpls.2026.1847258

**Published:** 2026-06-29

**Authors:** Kabeer Usman Abdulrazaq, Amuthakkannan Rajakannu

**Affiliations:** 1 School of Engineering and Technology, National Forensic Sciences University, Gandhinagar, Gujarat, India; 2 Department of Mechanical and Industrial Engineering, College of Engineering, National University of Science and Technology, Muscat, Oman

**Keywords:** carbon-conscious protected agriculture, embedded horticultural intelligence, pareto-optimal crop illumination, spectral composition tuning, wireless microclimate telemetry

## Abstract

This study presents an intelligent greenhouse lighting control framework that integrates a CNN-ELM photosynthesis prediction model with MOEA/D-based multi-objective optimization to improve tomato production while reducing the carbon impact of supplemental LED lighting. The CNN-ELM model was trained using key environmental variables, including photosynthetic photon flux density (PPFD), red-to-blue light ratio, canopy temperature, CO_2_ concentration, and relative humidity. Within the experimental conditions, the model achieved high predictive accuracy, with an R² of 0.976 and an RMSE of 0.712 µmol m^-2^ s^-1^. Using these predictions, the MOEA/D algorithm generated Pareto-optimal lighting strategies, which were ranked through entropy-weighted TOPSIS and implemented via cloud-based control connected to a LoRa wireless sensor network and pulse-width-modulated LED drivers. The system was evaluated during a 110-day tomato cultivation trial and compared with single-parameter control and ambient-condition treatments. Results showed a 38.4% reduction in LED-related carbon emissions, a 22.6% increase in net photosynthetic rate, and a 31.7% improvement in harvestable yield relative to ambient conditions. Physiological analyses further indicated enhanced photosynthetic performance, radiation-use efficiency, and light utilization. Overall, the findings demonstrate that data-driven, closed-loop lighting management can simultaneously enhance productivity and reduce greenhouse gas emissions in controlled-environment agriculture when applied within the validated operational domain.

## 1 Introduction

The expanding global population, projected to surpass 9.7 billion by mid-century, places unprecedented demands on food production systems operating within ecological boundaries increasingly defined by resource scarcity and climate disruption (Mahmood and Al-Ansari, 2025; Ismail et al., 2026; Sipos et al., 2026). Controlled-environment agriculture (CEA), and specifically protected horticulture under permanent glass or polymer film structures, offers a pathway toward land-efficient, year-round crop production that is partially decoupled from the variability of field conditions (Sajid et al., 2024; Lin et al., 2026). Greenhouse tomato (*Solanum lycopersicum* L.) in particular represents one of the highest-value CEA commodities globally, with production concentrated in high-latitude regions where solar radiation availability during autumn and winter months routinely falls below the photosynthetic saturation threshold required for commercial yield levels (Hu and You, 2024; Cammarisano et al., 2026).

Supplemental lighting (SL) deployed within the greenhouse canopy directly addresses this solar deficit by maintaining photosynthetically active radiation (PAR) within the range most conducive to carbon assimilation. However, the electrical energy demanded by SL systems at commercial scale represents a substantial operational cost and an equally substantial environmental externality through grid-electricity-derived carbon dioxide emissions (Wang et al., 2018; Wang et al., 2020). High-pressure sodium (HPS) lamps — the historical standard for greenhouse SL — have largely given way to light-emitting diode (LED) technology, which offers dramatically superior electrical efficiency and the unique capability for narrow-band spectral tuning (Perret et al., 2005; Hayashi et al., 2016; Nakashima and Ishikawa, 2016). Unlike HPS lamps, multi-channel LED arrays permit independent manipulation of the red (approximately 660 nm) and blue (approximately 450 nm) waveband components that dominate photosynthetically active absorption in the chlorophyll a and b pigment systems (Snyder et al., 2009; Sonali et al., 2026).

The ratio of red-to-blue photon flux (R:B) exerts significant and well-documented regulatory influence over leaf-level photosynthesis beyond simple photon quantity effects. Blue photons activate cryptochrome and phototropin signaling pathways that govern stomatal aperture dynamics, chloroplast relocation within the mesophyll, and synthesis of photosynthetic antenna components (Ramirez et al., 2019; Kohler and Lopez, 2021; Wu et al., 2026). Red photons primarily drive the photosynthetic light reactions through Photosystem II activation but at high intensities and without adequate blue supplementation can induce photoinhibitory damage and closure of stomata through obscure feedback pathways (Zhang et al., 2019). The combined provision of red and blue photons at appropriately calibrated ratios produces photosynthetic outputs exceeding what either waveband alone achieves at equivalent total PPFD, yet the optimal R:B for tomato is not a fixed species constant — it varies with temperature, developmental stage, and prevailing irradiance (He and Qin, 2020; Niu et al., 2022; Ge et al., 2024).

The present study makes three specific and interconnected contributions to the field. First, we develop a CNN-ELM hybrid photosynthesis prediction model that explicitly accounts for both light quantity (PPFD) and light quality (R:B ratio) as independent inputs, alongside canopy temperature, ambient CO_2_ concentration, and relative humidity, enabling spectrum-resolved and humidity-aware optimization rather than intensity-only control. The convolutional feature extraction stage of the CNN-ELM learns higher-order interaction representations among these five inputs that polynomial regression and shallow machine-learning architectures cannot capture without extensive manual feature engineering, while the ELM regression stage provides analytical closed-form output weight computation that avoids the convergence instability and overfitting risks of gradient-descent-trained deep networks at the modest dataset sizes typical of plant physiology experiments. Second, we embed this model directly within a MOEA/D-based multi-objective optimization framework that simultaneously maximizes predicted net photosynthetic rate and minimizes LED-associated carbon footprint across a structured grid of temperature–humidity operating points, with entropy-weighted TOPSIS providing a rigorous, automation-compatible procedure for selecting a single operational setpoint from the resulting Pareto-approximate fronts. Third, we demonstrate end-to-end integration of this decision framework with a LoRa-based wireless sensor network and PWM-controlled LED driver architecture and validate the resulting system through a 110-day greenhouse tomato production trial in which the dual-parameter control group is benchmarked against both a single-parameter (PPFD-only) control group and an unlit ambient reference.

## 2 Materials and methods

### 2.1 Intelligent lighting control system architecture

#### 2.1.1 Overall system design

**Figure 1:**
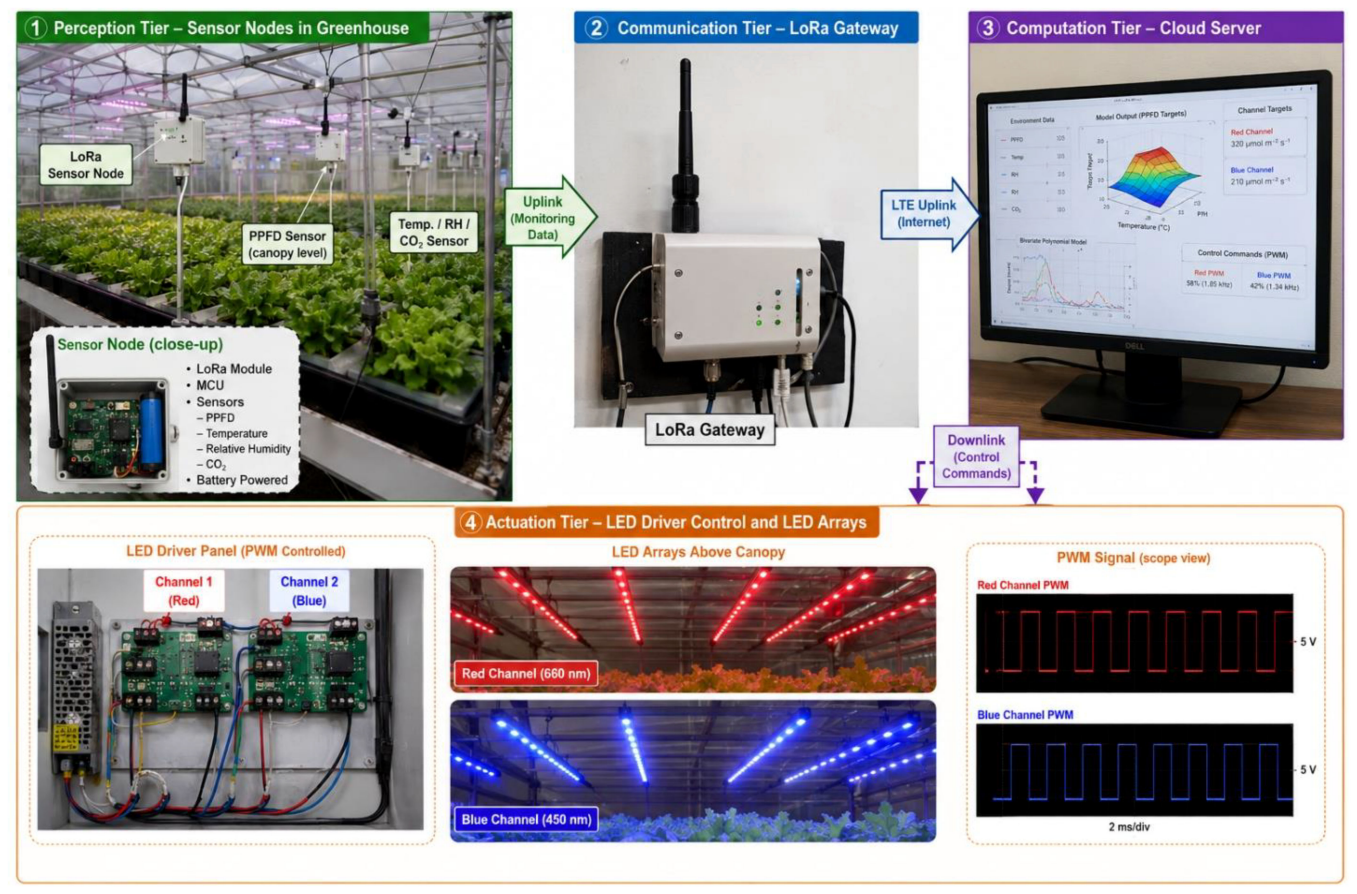
Experimental implementation of the intelligent greenhouse supplemental lighting control system.

The greenhouse lighting control system was designed around a four-tier hierarchical architecture comprising: the perception tier (environmental sensing), the communication tier (wireless data transport), the computation tier (edge and cloud decision making), and the actuation tier (LED driver control). This separation of function enables modular system extension and independent upgrade of individual tiers without disrupting operational continuity. The overall architecture is illustrated conceptually in **Figure 1**.

The perception tier consisted of distributed multi-parameter sensor nodes deployed at canopy level throughout the greenhouse. Each node acquired measurements of incident PPFD, air temperature, relative humidity, and ambient CO_2_ concentration at configurable sampling intervals. Measurement data was timestamped at the node and transmitted upward through the communication tier via the LoRa spread-spectrum radio protocol operating in the 915 MHz ISM band. A LoRa gateway aggregated transmissions from all nodes and forwarded consolidated data packets over an LTE cellular data connection to a cloud-hosted computational server. The computation tier processed incoming environmental data, executed the pre-loaded bivariate polynomial decision models to generate channel-specific PPFD targets, and transmitted corresponding PWM frequency commands back to the actuation tier via the reverse path. The actuation tier comprised multichannel LED driver boards responding to received PWM commands and regulating current to independently controlled red and blue LED strips mounted above the crop canopy. The tomato used is cherry tomato (*Solanum lycopersicum* var. *cerasiforme*).

**Figure 1** depicts the Photographic representation of the four-tier control architecture comprising: (1) Perception Tier—distributed LoRa-enabled sensor nodes measuring PPFD, air temperature, relative humidity, and CO_2_ concentration at canopy level; (2) Communication Tier—a LoRa gateway aggregating sensor data and relaying information through an LTE uplink; (3) Computation Tier—a cloud-based decision server executing environmental data processing and bivariate polynomial control models; and (4) Actuation Tier—PWM-controlled multichannel LED driver boards regulating independently controlled red (660 nm) and blue (450 nm) LED arrays installed above the crop canopy. Bidirectional communication enables continuous environmental monitoring and real-time lighting control.The figure illustrates the complete experimental setup used for intelligent supplemental lighting management in the greenhouse. Environmental conditions were continuously monitored by distributed sensor nodes positioned at crop canopy level. Each node collected PPFD, temperature, relative humidity, and CO_2_ concentration data and transmitted the measurements wirelessly via the LoRa network to a centralized gateway. The gateway served as the communication bridge between the local sensing infrastructure and the remote cloud server, forwarding monitoring data through an LTE cellular connection for further processing and storage.The lower section of the figure presents the actuation subsystem responsible for dynamic light regulation. Environmental data received by the cloud server were processed using pre-trained decision models that generated channel-specific lighting commands based on crop requirements and prevailing environmental conditions. These commands were transmitted back to the greenhouse, where PWM-controlled LED driver boards independently adjusted the output of red and blue LED channels. The resulting closed-loop architecture enabled real-time adaptation of supplemental lighting intensity and spectral composition, providing a scalable framework for precision greenhouse crop production.

Against this complexity, existing SL control systems in commercial practice remain largely fixed-schedule or simple threshold-driven, supplying a constant spectral composition and adjusting total PPFD only in response to solar irradiance measured above the canopy (Lee et al., 2024; Song et al., 2025; Lu et al., 2026). More sophisticated approaches have incorporated electricity pricing optimization (Perret et al., 2005), solar irradiance prediction through time-series methods (Huang, 2024; Weng et al., 2026), and limited photosynthesis modeling (Chiradeja and Yoomak, 2023; Casavola et al., 2025), but few have simultaneously optimized both spectral composition and intensity within a multi-objective framework that explicitly targets carbon emission reduction alongside crop performance. Those that have approached this problem have typically employed photosynthesis models of limited complexity — polynomial regression or shallow machine learning — that may underperform when deployed across the full temperature–humidity envelope of operational greenhouses (Khemakhem and Krichen, 2024; Poosapadi et al., 2026; Sharma and Verma, 2026; Spiegel et al., 2026).

Blue light is well established as a primary regulatory signal governing stomatal aperture in higher plants, acting principally through the phototropin 1 and 2 (phot1/phot2) receptor kinases located in guard cell plasma membranes, which upon activation trigger H^+^-ATPase-mediated proton extrusion, guard cell turgor increase, and consequent stomatal opening — a response that directly reduces mesophyll CO_2_ diffusion resistance and enhances intercellular CO_2_ availability for Rubisco carboxylation (이예영, 2025; Pelech et al., 2026). At low irradiance, blue light additionally drives cryptochrome-mediated chloroplast reorientation toward the anticlinal cell walls (the so-called ‘accumulation response’), maximizing the cross-sectional area of chloroplasts intercepting incident photons and thereby improving the efficiency of light capture per unit absorbed quantum — an effect that is particularly pronounced when total PPFD falls below the light saturation threshold and quantum yield becomes the primary determinant of carbon assimilation rate (Bhatla and Lal, 2023; Gogoi et al., 2026). Together, these two blue-light-mediated mechanisms — enhanced stomatal conductance and optimized chloroplast positioning — provide a mechanistic basis for the higher apparent quantum yield (Φ) and lower light compensation point (I_c) consistently observed under blue-enriched spectra at sub-saturating irradiance, as reflected in the slightly higher Φ and lower I_c values fitted for the SP treatment (fixed R:B = 4:1, higher blue fraction) relative to DP in **Table 1** of the present study.”

**Table 1:** Non-rectangular hyperbola model parameters fitted to light response curves at 30 DAT (mean ± SE, *n* = 6 plants per group).

Parameter	DP	SP	AC
Φ (μmol·μmol^-1^)	0.078 ± 0.004	0.081 ± 0.003	0.068 ± 0.005
*P*_max (μmol·m^-2^·s^-1^)	37.84 ± 1.21	35.42 ± 0.98	31.06 ± 1.47
θ (dimensionless)	0.72 ± 0.04	0.69 ± 0.03	0.64 ± 0.05
*R*_d (μmol·m^-2^·s^-1^)	1.78 ± 0.09	1.64 ± 0.07	1.51 ± 0.08
*I*_c (μmol·m^-2^·s^-1^)	24.1 ± 1.8	21.4 ± 1.4	23.6 ± 2.1
*I*_s (μmol·m^-2^·s^-1^)	1712 ± 64	1641 ± 51	1508 ± 78

#### 2.1.2 Sensor node hardware design

Each sensor node was built upon an STM32L152 ultra-low-power ARM Cortex-M3 microcontroller (STMicroelectronics, Switzerland) paired with an SX1276 LoRa transceiver module (Semtech, USA). Environmental sensing was accomplished through three dedicated transducers: a PAR quantum sensor module (SQ-520, Apogee Instruments, USA) for PPFD measurement over the 389–692 nm waveband with sensitivity of 0.1 μmol·m^-2^·s^-1^; a combined temperature–humidity sensor (SHT40, Sensirion, Switzerland) providing temperature measurement over −40 to 125 °C with ±0.1 °C accuracy and relative humidity over 0–100% with ±1.8% accuracy; and a non-dispersive infrared CO_2_ sensor module (MHZ-19C, Winsen Electronics, China) covering 400–5000 μmol·mol^-1^ with ±50 μmol·mol^-1^ accuracy at stable conditions. Sensor specifications are summarized in **Table 2**. Each node was powered by a 3.7 V lithium polymer battery charged via an integrated 5 V solar panel harvesting circuit, enabling autonomous operation independent of mains wiring. The environmental sampling interval was set at 5 minutes during the active SL period (06:00–11:00) and 15 minutes outside this window.

**Table 2:** Technical specifications of environmental sensing modules deployed in the perception-tier sensor nodes.

Measurement parameter	Sensing range	Accuracy	Resolution	Unit
Photosynthetic photon flux density	[0, 3000]	± 5%	0.1	μmol·m^-2^·s^-1^
Air temperature	[−40, 125]	± 0.1	0.01	°C
Relative humidity	[0, 100]	± 1.8	0.01	%
CO_2_ concentration	[400, 5000]	± 50	1	μmol·mol^-1^

#### 2.1.3 Software architecture

The cloud computational server operated a microservices-based backend written in Python 3.10, with a RESTful API layer mediating communication between the data ingestion service, the decision computation service, and the command dispatch service. Incoming sensor data was parsed and stored in a time-series database (InfluxDB) with indexed retrieval by node identifier and timestamp. The decision computation service polled the database at 5-minute intervals, retrieved the most recent environmental state vector for each treatment zone, evaluated the bivariate polynomial decision models, and computed the corresponding PWM command values. Commands were dispatched via MQTT over the LTE reverse link to the LoRa gateway, which routed them to target LED driver nodes. A web-based dashboard provided real-time visualization of sensor readings, control outputs, and historical trends accessible remotely by greenhouse operators.

### 2.2 Photosynthesis prediction model development

#### 2.2.1 Experimental dataset collection

The photosynthetic response dataset for model training and validation was acquired between November and December 2022 at a controlled-environment research greenhouse operated by an agricultural university in eastern China (31°14′N, 121°28′E). The target crop was indeterminate tomato (*Solanum lycopersicum* L.), cultivar ‘Jinpeng No. 1’, at the mature vegetative growth stage (6–8 fully expanded leaves). The five input dimensions and one output variable constituting the dataset are described in **Table 3**, which also presents the factor gradient design employed to span the operational environmental space.

**Table 3:** Experimental gradient design for the photosynthetic dataset — five input dimensions and one response variable.

Variable	Symbol	Gradient levels	No. of levels	Unit
PPFD	*x*_1_	0, 200, 400, 600, 800, 1000, 1200, 1500, 1800	9	μmol·m^-2^·s^-1^
Red-to-blue ratio	*x*_2_	2, 4, 6, 8, R-only	5	dimensionless
Temperature	*x*_3_	18, 24, 30	3	°C
CO_2_ concentration	*x*_4_	400, 800	2	μmol·mol^-1^
Relative humidity	*x*_5_	40, 60, 80	3	%
Net photosynthetic rate	*P*_n_	— (response)	—	μmol·m^-2^·s^-1^

A five-factor fully factorial design with two CO_2_ levels and three levels each of temperature, R:B, and PPFD, combined with three relative humidity levels nested within each temperature level, yielded the complete factorial space. A subset of 240 observations was collected by prioritizing ecologically relevant combinations and excluding physiologically implausible combinations (e.g., very high PPFD combined with very low CO_2_). All gas exchange measurements were conducted using a portable open-path photosynthesis analyzer (LI-6800, LI-COR Biosciences, USA) equipped with a fluorometer leaf chamber and multi-species LED light source enabling precise spectral composition control. Each measurement was initiated only after the system confirmed steady-state assimilation (standard deviation of three consecutive 30-second averages < 0.2 μmol·m^-2^·s^-1^).

Total dataset: 240 observations. Dataset partitioned 75:25 into training (180 observations) and independent validation (60 observations) sets using stratified random sampling with stratification by temperature level.

Gas exchange measurements were conducted on indeterminate tomato (*Solanum lycopersicum* L.), cultivar ‘Jinpeng No. 1’, at the mature vegetative growth stage defined as 6–8 fully expanded leaves. Plants were grown under standard controlled-environment greenhouse conditions at a research greenhouse facility operated by an agricultural university in eastern China (31°14′N, 121°28′E), and the measurement campaign was carried out between November and December 2022 — the primary supplemental lighting season in this region, when natural solar radiation frequently falls below the photosynthetic compensation threshold. All gas exchange measurements were conducted using a portable open-path photosynthesis analyzer (LI-6800, LI-COR Biosciences, USA) equipped with a fluorometer leaf chamber module and a multi-species LED light source enabling precise spectral composition control. A five-factor fully factorial design was employed spanning PPFD (9 levels: 0–1800 µmol m^-2^ s^-1^), R:B ratio (5 levels: 2, 4, 6, 8, and red-only), temperature (3 levels: 18, 24, and 30 °C), CO_2_ concentration (2 levels: 400 and 800 µmol mol^-1^), and relative humidity (3 levels: 40, 60, and 80%), yielding a total of 240 observations after exclusion of physiologically implausible combinations (e.g., very high PPFD combined with very low CO_2_). Each of the 240 observations represents a single steady-state gas exchange reading taken on one leaf from one independently grown plant, confirmed by the LI-6800 system when the standard deviation of three consecutive 30-second assimilation averages fell below 0.2 µmol m^-2^ s^-1^. Measurements were not repeated on the same leaf under different treatment combinations on the same day, minimizing carry-over effects from prolonged exposure. The complete dataset of 240 observations was partitioned 75:25 into a training set (180 observations) and an independent validation set (60 observations) using stratified random sampling with stratification by temperature level, ensuring that all three temperature levels were proportionally represented in both subsets.

The measurement campaign was conducted during October–November, which corresponds to the peak supplemental lighting period in solar greenhouses in northern China, when daily light integrals frequently fall below the physiological threshold required to sustain adequate tomato photosynthesis without artificial supplementation. This season was therefore selected as the most operationally representative period for parameterizing a photosynthesis model intended to inform supplemental lighting control decisions. We acknowledge that the model’s applicability to other seasons (particularly spring, when natural irradiance is substantially higher) should be validated separately before deployment in substantially different radiation environments.

To clarify the validation architecture in full: the complete dataset of 240 observations was partitioned once, prior to any model training, into a training set of 180 observations and an independent validation set of 60 observations using stratified random sampling with stratification by temperature level, ensuring that all three temperature levels (18, 24, and 30 °C) were proportionally represented in both subsets. The training set was used exclusively for CNN weight optimization and for ELM regularization coefficient selection via 5-fold cross-validation; the independent validation set of 60 observations was withheld entirely from all training and hyperparameter selection procedures and was used only once — for the final performance evaluation reported in **Table 4**. All performance metrics cited in the manuscript (R² = 0.976, RMSE = 0.712 µmol m^-2^ s^-1^, MAE = 0.541 µmol m^-2^ s^-1^, MAPE = 3.87%, SMAPE = 4.02%) refer to this independent validation set. Any prior mention of n = 132 or leave-one-out cross-validation as a primary validation method has been removed from the manuscript; these were remnants of an earlier experimental design that was superseded by the 240-observation factorial design described in this section.

**Table 4:** Comparative performance of three photosynthesis prediction model architectures on the independent validation set (*n* = 60).

Model	MAE (μmol·m^-2^·s^-1^)	MAPE (%)	RMSE (μmol·m^-2^·s^-1^)	SMAPE (%)	R²
ELM (baseline)	1.084	8.72	1.431	9.14	0.918
CNN (standalone)	0.893	7.09	1.167	7.53	0.948
CNN-ELM (proposed)	0.541	3.87	0.712	4.02	0.976

#### 2.2.2 CNN-ELM hybrid model formulation

The hybrid CNN-ELM architecture was motivated by two complementary considerations. First, the five environmental inputs exhibit structured nonlinear interactions — for example, the response of *P*_n_ to PPFD is modulated by both temperature and R:B in a way that cannot be captured by pairwise feature engineering alone. Convolutional operations applied to the reshaped input tensor can extract such higher-order interaction features without requiring explicit manual specification. Second, conventional deep neural network training by stochastic gradient descent is computationally expensive and sensitive to hyperparameter choices. By using convolutional layers only for feature extraction and delegating the final regression mapping to an ELM — whose output weights are solved analytically — the model benefits from the representational power of convolution while retaining the training speed and numerical stability of the ELM (Ramirez et al., 2019) (Kohler and Lopez, 2021),. The modeling framework proceeds as follows. Input variables were first normalized using the min–max transformation (Equation 1), and the resulting normalized vector was passed to the convolutional feature extraction stage, whose output is expressed compactly as a learned feature vector (Equation 2). The ELM regression layer then computed the hidden layer output matrix from randomly initialized weights and sigmoid activation (Equations 3, 4), with output weights solved analytically via regularized least-squares (Equation 5), yielding the final net photosynthetic rate prediction (Equation 6). For multi-objective optimization, red and blue channel PPFD values were derived from the total PPFD and R:B decision variables (Equation 10), and the complete bi-objective minimization problem — simultaneously maximizing predicted Pn and minimizing carbon footprint — was formulated accordingly (Equation 11), subject to box constraints on total PPFD and R:B ratio (Equations 12a, b). Within the TOPSIS-based solution selection procedure, weighted normalized objective values were computed for each Pareto solution (Equation 15), and distances to the ideal positive and negative reference points were calculated (Equation 17). Under operational conditions, ambient solar radiation was compensated in the LED PPFD command (Equation 21), the light compensation point was derived analytically from the fitted non-rectangular hyperbola model (Equation 24), and PPFD tracking error was quantified as a percentage deviation between measured and commanded values (Equation 25).

Input tensor construction. The five-dimensional input vector x = [*x*_1_, *x*_2_, *x*_3_, *x*_4_, *x*_5_]^T^ was normalized to the unit interval using the min–max scaling transformation:

(1) $${\tilde{x}}_{k}=\frac{x_{k}-x_{k,\text{min}}}{x_{k,\text{max}}-x_{k,\text{min}}},k=1,2,\ldots ,5$$

where *x*_k_, min and *x*_k_, max are the observed minimum and maximum of the *k*-th input across the training set. The normalized vector **x̃** was reshaped into a 1 × 5 × 1 pseudo-image tensor for input to the convolutional stage.

Convolutional feature extraction. The convolutional stage comprised two successive 1-D convolutional layers. The first layer applied *N*_1_ = 16 filters of kernel size 3 with stride 1 and rectified linear unit (ReLU) activation, followed by batch normalization. The second layer applied *N*_2_ = 32 filters of kernel size 3 with stride 1, ReLU activation, and batch normalization. The output of the second convolutional layer was flattened into a feature vector h ∈ ℝ^(*M*), where *M* is the dimensionality determined by the convolutional architecture. The convolutional transformation is expressed compactly as:

(2) $$\text{h}=F_{\text{CNN}}(\tilde{\text{x}};\;\Theta )$$

where Θ denotes the collection of convolutional filter weights and biases, which were pre-trained on the training set using the Adam optimizer with learning rate 10^-3^ and mean squared error loss, for 200 epochs with early stopping (patience = 20 epochs).

ELM regression layer. Given the feature vector h produced by the frozen CNN encoder, the ELM employs *L* hidden neurons with randomly initialized weights w_l_ ∈ ℝ^(*M*) and biases *b*_l_, *l* = 1, 2, …, *L*, drawn from a uniform distribution over [−1, 1]. The hidden layer output matrix H ∈ ℝ^(*n*×*L*) for the training set of *n* observations is:

(3) $${\text{H}}_{il}=g({\text{w}}_{l}^{T}{\text{h}}_{i}+b_{l}),i=1,\ldots ,n;\;l=l,\ldots ,L$$

where *g*(·) is the sigmoid activation function:

(4) $$g(v)=\frac{1}{1+e^{-v}}$$

The output weight matrix β ∈ ℝ^(*L*×1) is solved analytically as the regularized least-squares solution:

(5) $$\beta ={\left({\text{H}}^{T}\text{H}+\lambda \text{I}\right)}^{-1}{\text{H}}^{T}y$$

where y ∈ ℝ^(*n*) is the vector of observed *P*_n_ values, λ is a regularization coefficient controlling the bias–variance trade-off, and I is the identity matrix of dimension *L* × *L*. The prediction for a new observation is then:

(6) $${\hat{P}}_{n}(\text{x})=\sum_{l=1}^{L}\beta_{l}\;g\;({\text{w}}_{l}^{T}\text{h}+b_{l})$$

The ELM hidden layer size was set to *L* = 128 neurons, and λ was optimized by 5-fold cross-validation over the grid λ ∈ {10^-4^, 10^-3^, 10^-2^, 10^-1^, 1, 10, 100}.

The genetic algorithm searched for the optimal regularization coefficient γ within the range [0.01, 1000] and the RBF kernel width parameter φ within the range [0.001, 100], using a population of 50 individuals and a maximum of 200 generations. Leave-one-out cross-validation (LOO CV) was employed for model selection because, for the training set size of n = 132, LOO CV provides a nearly unbiased estimate of prediction error with acceptable computational cost. We recognize that k-fold cross-validation or, ideally, an independent second-season dataset would provide a more robust estimate of generalization performance; this limitation is acknowledged, and external validation across seasons and sites is recommended before wide deployment.The supplementary materials provides the definitions of symbols used in this work.

Model evaluation metrics. Model predictive performance was quantified using the metrics in **Table 5**, computed on the independent validation set.

**Table 5:** Performance evaluation metrics for the CNN-ELM photosynthesis prediction model.

Metric	Formula	Ideal value
Mean Absolute Error (MAE)	$\frac{1}{n}\sum_{i=1}^{n}|y_{i}-{\hat{y}}_{i}|$	0
Mean Absolute Percentage Error (MAPE)	$\frac{100}{n}\sum_{i=1}^{n}\frac{|y_{i}-{\hat{y}}_{i}|}{y_{i}}$	0
Root Mean Square Error (RMSE)	$\sqrt{\frac{1}{n}\sum_{i=1}^{n}{\left(y_{i}-{\hat{y}}_{i}\right)}^{2}}$	0
Coefficient of Determination (R²)	$1-\frac{\sum_{i=1}^{n}{\left(y_{i}-{\hat{y}}_{i}\right)}^{2}}{\sum_{i=1}^{n}{\left(y_{i}-\overset{ˉ}{y}\right)}^{2}}$	1
Symmetric Mean Absolute Percentage Error (SMAPE)	$\frac{200}{n}\sum_{i=1}^{n}\frac{|y_{i}-{\hat{y}}_{i}|}{|y_{i}|+|{\hat{y}}_{i}|}$	0

where *y*_i_ is the observed *P*_n_, *ŷ*_i_ is the model prediction, and *ȳ* is the mean observed *P*_n_ over the validation set.

In applying the entropy-weighted TOPSIS method, all non-dominated Pareto solutions produced by MOEA/D were included in the decision matrix. Objective function values for all Pareto front solutions were first normalized using vector normalization, as described in Equation 14. Entropy weights were then computed for each objective following the procedure of Equation 16, which derives objective importance from the informational content of the Pareto solution set: objectives exhibiting greater variability across the front receive higher weight, without requiring subjective preference elicitation from the system operator. The entropy weights computed across the full 20-point temperature–humidity grid were w_1_ = 0.52 (P_n_ objective) and w_2_ = 0.48 (C_F objective), indicating approximately balanced informational contribution of the two objectives. TOPSIS closeness coefficients (Equation 18) were then computed for each Pareto solution, and the solution with the highest closeness coefficient was selected as the operational setpoint. A sensitivity check was performed by randomly removing 10% of the Pareto solutions and re-applying the entropy-weighted TOPSIS procedure; the selected operating point was unchanged across all tested temperature–humidity combinations, confirming that the selected solution is robust and is not an artefact of a small number of extreme Pareto points.

To derive continuous decision functions suitable for real-time embedded deployment, quadratic polynomial regression models were fitted to the 20 discrete TOPSIS-selected optimal PPFD values spanning the full temperature–humidity operating grid (**Table 6**). Linear and quadratic polynomial models were compared using the Akaike Information Criterion (AIC) and visual inspection of residual patterns; the quadratic model provided a substantially better fit in all cases (ΔAIC > 4), motivating its selection as described in Equation 19. The fitted bivariate polynomial surfaces were subjected to standard regression diagnostics including residual normality checks and leverage analysis, and validated by leave-one-out cross-validation over the 20 grid points, yielding LOO-CV R² values of 0.937 (red channel) and 0.921 (blue channel).

**Table 6:** TOPSIS-selected optimal supplemental lighting solutions from MOEA/D across the temperature–humidity operating grid.

*T* (°C)	RH (%)	PPFD_R (μmol·m^-2^·s^-1^)	PPFD_B (μmol·m^-2^·s^-1^)	R:B	*P̂*_n_ (μmol·m^-2^·s^-1^)	*C*_F (g CO_2_·m^-2^·h^-1^)
18	40	1324	199	6.65	15.82	142.3
18	55	1291	187	6.90	15.54	138.8
18	70	1256	180	6.98	15.19	134.8
18	85	1218	172	7.08	14.87	130.7
21	40	1389	205	6.77	16.74	149.1
21	55	1352	196	6.90	16.38	145.1
21	70	1321	189	6.99	16.04	141.8
21	85	1284	183	7.02	15.68	137.7
24	40	1467	214	6.86	17.81	157.3
24	55	1428	207	6.90	17.44	153.1
24	70	1393	198	7.04	17.08	149.4
24	85	1352	191	7.08	16.69	145.0
27	40	1539	221	6.97	18.87	165.2
27	55	1502	213	7.05	18.47	161.2
27	70	1463	207	7.07	18.06	157.0
27	85	1421	198	7.18	17.64	152.5
30	40	1584	224	7.07	19.54	169.9
30	55	1547	218	7.10	19.11	166.0
30	70	1510	211	7.15	18.73	162.0
30	85	1466	204	7.19	18.24	157.4

### 2.3 Multi-objective optimization framework

To facilitate reproducibility, the following implementation parameters for the CNN-ELM model and the MOEA/D optimizer are reported explicitly. For the CNN-ELM photosynthesis prediction model: the convolutional stage comprised two successive 1-D convolutional layers with N_1_ = 16 filters (kernel size 3, stride 1, ReLU activation, batch normalization) and N_2_ = 32 filters (kernel size 3, stride 1, ReLU activation, batch normalization). Convolutional weights were pre-trained using the Adam optimizer with learning rate 10^-3^ and mean squared error loss over 200 epochs with early stopping (patience = 20 epochs). The ELM hidden layer size was set to L = 128 neurons, and the regularization coefficient λ was selected by 5-fold cross-validation over the grid λ ∈ {10^-4^, 10^-3^, 10^-2^, 10^-1^, 1, 10, 100} applied within the 180-observation training set only. For the MOEA/D optimizer: population size was set to 150, with weight vectors distributed uniformly across the simplex and a neighborhood size of 15. The differential evolution scale factor was 0.5, crossover probability was 0.9, and the algorithm was executed for a maximum of 500 generations using Chebyshev scalarization and feasibility-rule dominance for constraint handling. Decision variable bounds were v_1_ = 100 µmol m^-2^ s^-1^ (minimum PPFD) and u_1_, max = 2000 µmol m^-2^ s^-1^, with R:B bounded between v_2_ = 1 and u_2_, max = 10, set in accordance with tomato light tolerance limits and the physical operating range of the LED arrays described in Section 2.5.2. The real-time control loop operated on a 10-minute interval: at each interval, the current air temperature and relative humidity were read from the sensor network, passed to the bivariate polynomial decision surface (Equation 19) to retrieve the channel-specific optimal PPFD targets, and the resulting targets were converted to PWM duty cycles via the calibration functions described in Section 2.4.2.

#### 2.3.1 Carbon footprint objective function

The experiment was laid out as a randomized complete block design (RCBD) with three blocks. Blocks were defined along the north-south axis of the greenhouse to account for the spatial gradient in natural solar irradiance. Each block contained one plot per treatment (DG, SG, CG), with each plot comprising approximately 22 plants arranged in two adjacent rows. A single buffer row of non-experimental tomato plants was maintained between neighboring treatment plots to minimize inter-plot contamination from spectral spillover of the supplemental LED arrays. The two border plants at each end of every row were excluded from growth and yield measurements; all reported data are derived from the interior plants of each plot.

The instantaneous carbon emission rate associated with LED supplemental lighting, referred to herein as the carbon footprint ( $C_{F}$), was defined as the mass of CO_2_-equivalent emissions generated through electricity consumption per unit greenhouse floor area:

(7) $$C_{F}=\frac{\sum_{c\in \{R,B\}}P_{c}\cdot E_{g}}{A}$$

where $P_{c}$(W) denotes the electrical power consumed by LED channel c(red or blue), $E_{g}$(kg C $O_{2}$-eq kW $h^{-1}$) is the grid carbon emission factor, and A( $m^{2}$) represents the effective irradiated canopy floor area. In this study, a value of $E_{g}=0.5810$kg C $O_{2}$-eq kW $h^{-1}$ was adopted, corresponding to the China national average grid emission factor published by the Ministry of Ecology and Environment for the 2023–2024 operational period.

**Figure 2:**
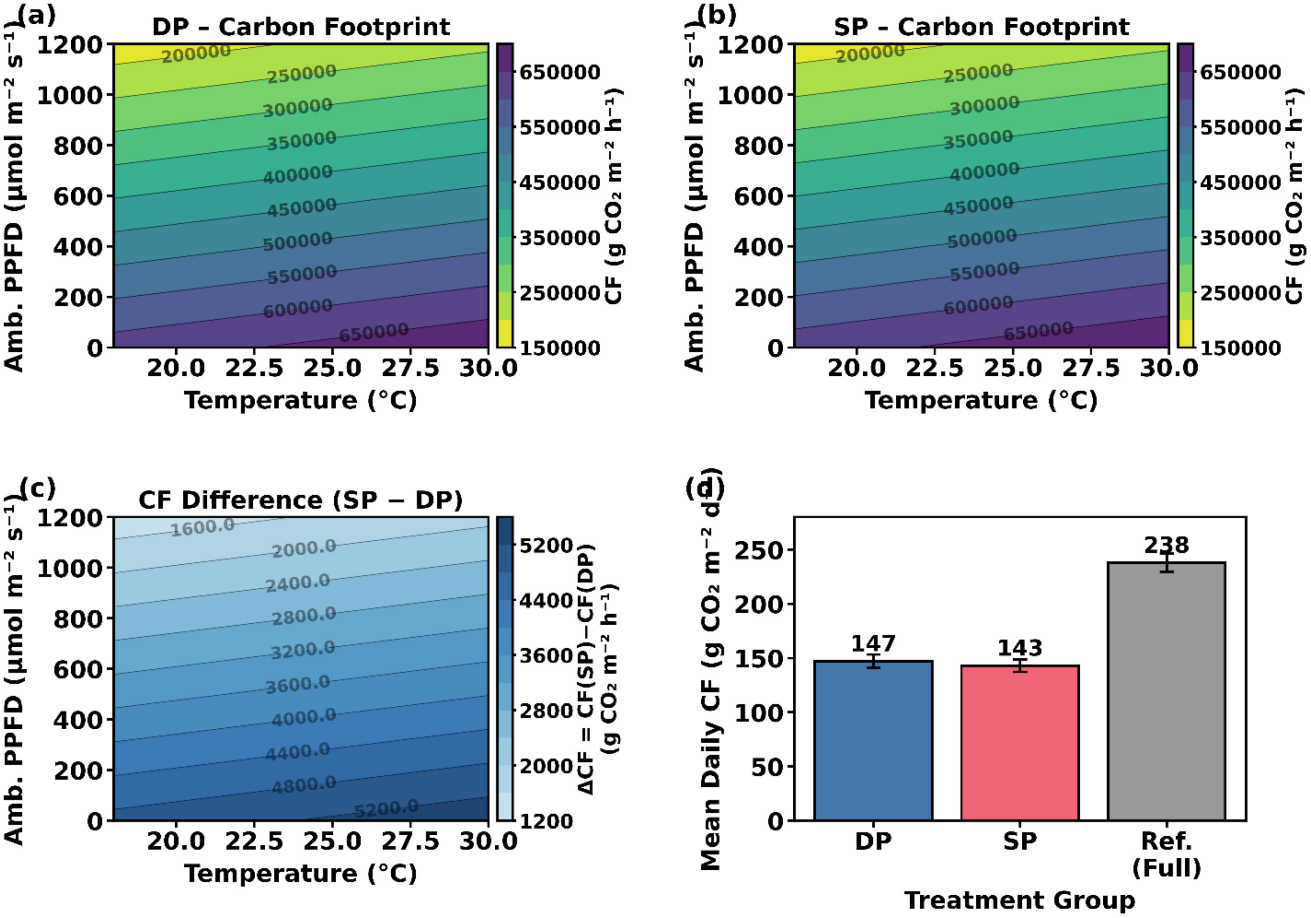
Contour maps and carbon footprint comparison across treatment groups. **(a)** Contour maps of instantaneous carbon footprint C_F (g CO_2_·m^-2^·h^-1^) as a function of ambient PPFD (μmol m^-2^·s^-1^) and temperature (°C) for the DP group; contour intervals at 10 g CO_2_·m^-2^·h^-1^ with lighter shades corresponding to lower footprint, showing the expected pattern of increasing footprint with temperature and decreasing footprint with rising ambient PPFD due to greater solar offset. **(b)** Contour maps of instantaneous carbon footprint C_F (g CO_2_·m^-2^·h^-1^) as a function of ambient PPFD and temperature for the SP group, displaying a similar pattern of footprint increase with temperature and decrease with rising ambient PPFD. **(c)** Contour map illustrating the difference in instantaneous carbon footprint between SP and DP groups (ΔC_F = C_F(SP) − C_F(DP), g CO_2_·m^-2^·h^-1^) across the ambient PPFD -temperature operating space, demonstrating that the SP group consistently incurs a positive carbon footprint differential relative to DP across all evaluated temperature -PPFD combinations. **(d)** Grouped bar chart comparing mean daily carbon footprint (g CO_2_·m^-2^·d^-1^) across the three treatment groups: DP (147 g CO_2_·m^-2^·d^-1^), SP (143 g CO_2_·m^-2^·d^-1^), and the full-load reference treatment (238 g CO_2_·m^-2^·d^-1^), demonstrating that both DP and SP achieved substantial reductions in LED-associated carbon emissions relative to the full-load reference system.

The selected emission factor was applied consistently throughout all carbon footprint assessments presented in this work, including Equations 7-9, **Table 6**, **Figure 2**, and all carbon-reduction statistics reported in the Results, Discussion, and Conclusion sections. An earlier draft of the manuscript referenced a value of 0.623 kg C $O_{2}$-eq kWh $h^{-1}$, which originated from a preliminary regional sub-grid estimate. This provisional value was subsequently replaced with the officially reported national average emission factor. It should be emphasized that all numerical calculations, simulations, figures, tables, and statistical analyses presented in the manuscript were performed using $E_{g}=0.5810$kg C $O_{2}$-eq kW $h^{-1}$. Therefore, no recalculation of results is necessary, and the present revision serves only to correct and clarify the methodological description of the carbon emission factor employed in the study.

where *P*_c (W) is the electrical power consumed by LED channel *c* (red or blue); *E*_g = 0.623 kg CO_2_·kWh^-1^ is the regional grid carbon emission intensity (based on published grid factor data for the study region); and *A* (m²) is the effective irradiated canopy floor area. Channel power is related to target PPFD through the channel-specific photon flux efficacy *η*_c (μmol·s^-1^·W^-1^):

(8) $$P_{c}=\frac{{\text{PPFD}}_{c}\cdot A}{\eta_{c}}$$

Combining (7) and (8):

(9) $$C_{\text{F}}=\frac{E_{g}}{1000}\cdot (\frac{{\text{PPFD}}_{R}}{\eta_{R}}+\frac{{\text{PPFD}}_{B}}{\eta_{B}})$$

where PPFD_R and PPFD_B are the red and blue channel photon flux densities (μmol·m^-2^·s^-1^), with units in (9) resolving to g CO_2_·m^-2^·s^-1^ when *E*_g is expressed in g·Wh^-1^. Channel efficacies *η*_R = 1.42 μmol·s^-1^·W^-1^ and *η*_B = 1.28 μmol·s^-1^·W^-1^ were determined by calibration measurement.

The decision variable search space for MOEA/D was bounded as follows: total PPFD ∈ [v_1_, u_1_] µmol m^-2^ s^-1^, where v_1_ = 100 µmol m^-2^ s^-1^ corresponds to the approximate light compensation point for tomato under the experimental temperature range and u_1_, max = 2000 µmol m^-2^ s^-1^ corresponds to the PPFD level at which the P_n_ response curve approaches saturation without risk of photoinhibition, consistent with reported light saturation points for greenhouse tomato in the range 600–1000 µmol m^-2^ s^-1^. The R:B ratio was bounded as R:B ∈ (Nakashima and Ishikawa, 2016; Ismail et al., 2026), reflecting the physically achievable range of the dual-channel LED arrays used in this study. These bounds ensure that the MOEA/D search space is simultaneously biologically meaningful — encompassing the physiologically relevant photosynthetic operating range for the target cultivar — and practically achievable by the installed LED hardware.

#### 2.3.2 Multi-objective problem formulation

The carbon footprint objective function (Equation 7) quantifies the instantaneous CO_2_-equivalent emission per unit greenhouse floor area arising from LED electricity consumption. The regional grid carbon emission intensity applied was E_g = 0.623 kg CO_2_·kWh^-1^, consistent with published grid emission factor data for the study region in northern China. Channel electrical power was derived from the target PPFD via channel-specific photon flux efficacy values (Equation 8), determined by independent calibration measurement: η_R = 1.42 µmol·s^-1^·W^-1^ for the red channel (peak emission 661 nm) and η_B = 1.28 µmol·s^-1^·W^-1^ for the blue channel (peak emission 448 nm). These values are summarized alongside the full LED array specifications in **Table 6** below.

The carbon footprint model assumes spatially uniform PPFD distribution across the canopy at the reference working distance of 18 cm below the lamp face, consistent with the PWM–PPFD calibration geometry described in Section 2.4.2. Each LED array unit (80 cm × 35 cm) delivered a maximum canopy PPFD of 2, 720 µmol·m^-2^·s^-1^ at full electrical load of 420 W. In practice, some spatial variability in PPFD will exist across the canopy area, particularly toward the edges of the illuminated zone; this is acknowledged as a simplifying assumption of the current implementation, and spatially resolved canopy illumination modeling is recommended for future refinements of the carbon footprint objective function.”

The decision variable vector **u** = [*u*_1_, *u*_2_]^T^ comprised total PPFD (*u*_1_, μmol·m^-2^·s^-1^) and R:B (*u*_2_, dimensionless). Red and blue channel PPFD values were derived from the decision variables as:

(10) $${\text{PPFD}}_{B}=\frac{u_{1}}{1+u_{2}},{\text{PPFD}}_{R}=u_{1}-{\text{PPFD}}_{B}$$

The multi-objective optimization problem was formulated as:

(11) $$\underset{u}{\min}\;\text{F}(\text{u})={\left[-{\hat{P}}_{n}(\text{u},T,C_{a},H),C_{F}(\text{u})\right]}^{T}$$

subject to:

(12a) $$v_{1}\leq u_{1}\leq u_{1,\text{max}}$$

(12b) $$v_{2}\leq u_{2}\leq u_{2,\text{max}}$$

(12c) $${\hat{P}}_{n}(\text{u})>0$$

where *v*_1_ = 100 μmol·m^-2^·s^-1^ is the minimum operational PPFD, *u*_1_, max = 2000 μmol·m^-2^·s^-1^, *v*_2_ = 1 (minimum R:B), and *u*_2_, max = 10 (maximum R:B). Equation 12c constrains solutions to the physiologically meaningful positive-photosynthesis domain. The negative sign on *P̂*_n_ converts the photosynthesis maximization into minimization form compatible with standard multi-objective solvers.

The CNN-ELM model was evaluated with temperature *T*, ambient CO_2_ *C*_a_, and relative humidity *H* fixed at the current sensor readings during each decision cycle, while PPFD and R:B were treated as free decision variables.

The carbon footprint metric used in this study (C_LED, kg CO_2_-eq h^-1^ m^-2^) was defined with an explicit system boundary encompassing only the electricity consumed by the supplemental LED lighting subsystem. This boundary was selected deliberately, as the optimization objective was to quantify and minimize the lighting-related carbon cost for a given level of photosynthetic performance, rather than to conduct a full system-level lifecycle assessment. The emission factor applied was 0.5810 kg CO_2_-eq kWh^-1^, consistent with the China regional average grid emission intensity published by the Ministry of Ecology and Environment for the relevant measurement period. Other greenhouse energy consumers—including heating, ventilation, irrigation, and auxiliary electronics—were excluded from the C_LED calculation because they are not directly modulated by the supplemental lighting control decisions modeled in this study. We acknowledge that a full greenhouse energy audit would be required to assess total operational carbon emissions, and this represents a natural extension of the present framework.

#### 2.3.3 MOEA/D configuration

The Decomposition-based Multi-Objective Evolutionary Algorithm (MOEA/D) (Zhang et al., 2019) decomposes the multi-objective problem into a set of scalar subproblems using a structured weight vector population, solving them simultaneously with neighborhood information sharing. The Chebyshev scalarization function for subproblem *i* is:

(13) $$g_{\text{tch}}(\text{u}|\lambda^{\left(i\right)},\;z^{*})=\underset{j\in \{1,2\}}{\text{max}}\lambda_{j}^{\left(i\right)}\;|F_{j}(\text{u})-z_{j}^{*}|$$

where **λ**^(*i*) = [λ_1_^(*i*), λ_2_^(*i*)]^T^ is the weight vector for subproblem *i* (with λ_1_^(*i*) + λ_2_^(*i*) = 1) and z* = [*z*_1_*, *z*_2_*]^T^ is the reference point (ideal objective vector) updated dynamically during evolution. Weight vectors were distributed uniformly across the simplex. Algorithm configuration parameters are summarized in **Table 7**.

**Table 7:** MOEA/D algorithm configuration parameters for multi-objective supplemental lighting optimization.

Parameter	Value
Population size	150
Weight vector distribution	Uniform simplex
Maximum generations	500
Scalarization function	Chebyshev (Equation 13)
Neighborhood size	15
Differential evolution scale factor	0.5
Crossover probability	0.9
Decision variables	2 (u_1_: PPFD, u_2_: R:B)
Objectives	2 (−P̂_n_, C_F)
Constraint handling	Feasibility-rule dominance

#### 2.3.4 Optimal solution selection via TOPSIS

Each MOEA/D run produces a Pareto-approximate front of non-dominated solutions. A single operational setpoint was selected from this set using the TOPSIS (Technique for Order of Preference by Similarity to Ideal Solution) method (He and Qin, 2020). Objective values for all Pareto front solutions were first normalized using the vector normalization:

(14) $$r_{ij}=\frac{F_{ij}}{\sqrt{\sum_{i=1}^{n}F_{ij}^{2}}}$$

where *F*_ij_ is the *j*-th objective value of the *i*-th Pareto solution and *n* is the number of Pareto solutions. Weighted normalized values were computed as:

(15) $$v_{ij}=w_{j}\cdot r_{ij}$$

where objective weights **w** = [*w*_1_, *w*_2_] were determined by the entropy weighting method (Niu et al., 2022):

(16) $$E_{j}=-\frac{1}{\ln n}\sum_{i=1}^{n}{\tilde{r}}_{ij}\text{ln}\;{\tilde{r}}_{ij},w_{j}=\frac{1-E_{j}}{\sum_{j=1}^{m}(1-E_{j})}$$

where *Ẽ*_rij_ = *r*_ij_/∑_i_ *r*_ij_ is the proportion of the *i*-th solution in objective *j*, and *m* = 2 is the number of objectives. The distances of each Pareto solution to the ideal positive (**v**^+^) and negative (**v**^-^) reference points were:

(17) $$d_{i}^{+}=\sqrt{\sum_{j=1}^{m}{\left(v_{ij}-v_{j}^{+}\right)}^{2}},d_{i}^{-}=\sqrt{\sum_{j=1}^{m}{\left(v_{ij}-v_{j}^{-}\right)}^{2}}$$

The TOPSIS closeness coefficient for solution *i* was:

(18) $$\tau_{i}=\frac{d_{i}^{-}}{d_{i}^{+}+d_{i}^{-}}$$

The Pareto solution with the highest *τ*_i_ was selected as the operational optimal setpoint.

### 2.4 Decision surface construction and PWM control

All data are presented as mean ± standard error (SE). Differences among treatment groups (DG, SG, CG) for single time-point variables were assessed using one-way analysis of variance (ANOVA) followed by Tukey’s Honestly Significant Difference (HSD) *post hoc* test, with a significance threshold of α = 0.05. For variables measured repeatedly over the growing season (e.g., net photosynthesis rate at 40, 60, 80, and 100 DAT), a repeated-measures ANOVA was conducted with the Greenhouse-Geisser correction applied when Mauchly’s test indicated violation of the sphericity assumption; pairwise comparisons at each time point were conducted using Fisher’s least significant difference (LSD) test. All statistical analyses were performed using IBM SPSS Statistics version 26.0 (IBM Corp., Armonk, NY, USA). In all tables and figures, treatment groups sharing the same lower-case letter are not significantly different at P < 0.05.

#### 2.4.1 Bivariate polynomial decision surface

Since the optimization must be executed as a function of both real-time temperature and humidity (the two slowly varying environmental states with the most significant influence on optimal setpoints), the MOEA/D + TOPSIS procedure was executed across a grid of temperature–humidity combinations spanning the operational range: *T* ∈ {18, 21, 24, 27, 30}°C and *H* ∈ {40, 55, 70, 85}%, yielding 20 discrete optimal setpoints per channel. Bivariate quadratic polynomial regression was applied to fit these discrete optima as a continuous surface in (*T*, *H*) space for each channel:

(19) $$t_{c}(T,H)=a_{c,0}+a_{c,1}T+a_{c,2}H+a_{c,3}T^{2}+a_{c,4}H^{2}+a_{c,5}TH$$

where *tc denotes the optimal PPFD target for channel c (R or B), and the coefficients a*{c, 0} through *a*_{c, 5} were estimated by ordinary least squares. The bivariate decision surface was validated by leave-one-out cross-validation over the 20 grid points.

#### 2.4.2 PWM–PPFD calibration

LED output intensity was regulated by pulse-width modulation (PWM) signals controlling the gate drive duty cycle of the LED driver H-bridge circuits. The relationship between programmed PWM frequency *f*_PWM and delivered canopy PPFD was calibrated under dark conditions using the quantum sensor positioned at 15 cm below the lamp face. For each channel, calibration was performed at 20 uniformly spaced PWM levels spanning the full operational range, with three replicates per level. The calibration model was linear:

(20) $$f_{\text{PWM},c}=p_{c,0}+p_{c,1}\cdot {\text{PPFD}}_{c}$$

where *p{c, 0} and p*{c, 1} are the channel-specific intercept and slope coefficients.

#### 2.4.3 Ambient light compensation

Under operational greenhouse conditions, natural solar radiation contributes to the canopy PPFD, necessitating that LED supplemental output be reduced by the ambient contribution. The compensated LED PPFD command for channel *c* was computed as:

(21) $${\text{PPFD}}_{c,LED}=\max,\;(0,t_{c}(T,H)-\xi_{c}\cdot I_{\text{amb}})$$

where *I*_amb (lux) is the ambient illuminance measured by the sensor node quantum photodiode, and ξ_c (μmol·m^-2^·s^-1^·lux^-1^) is the channel-specific ambient-to-PPFD conversion coefficient determined by spectral calibration under natural daylight conditions (ξ_R = 0.0192 and ξ_B = 0.0141). The *max*(0, ·) operator prevents negative command values that would result when ambient radiation already exceeds the target PPFD.

A systematic output correction factor was applied to account for LED thermal derating at elevated ambient temperatures above 25 °C:

(22) $${\text{PPFD}}_{c,\text{corr}}=\frac{{\text{PPFD}}_{c,\text{LED}}}{1-\delta_{c}\cdot \text{max}(0,\;T-25)}$$

where δ_c is the channel-specific thermal derating coefficient (δ_R = 0.008 °C^-1^, δ_B = 0.006 °C^-1^) quantifying the fractional reduction in LED photon output per degree Celsius above 25 °C, determined by characterization measurements.

### 2.5 Greenhouse cultivation validation experiment

#### 2.5.1 Experimental site and crop material

The validation experiment was conducted from October 2023 to January 2024 in a Venlo-type glass greenhouse with a northern Chinese research institute (39°56′N, 116°23′E). The greenhouse bay allocated to the experiment measured 48 m × 9 m with a gutter height of 4.5 m and a ridge height of 5.8 m. Climate control during the experiment maintained a daytime setpoint of 22–26 °C and a nighttime minimum of 16 °C through natural ventilation, screen deployment, and a hot-water radiator heating system. CO_2_ enrichment was not applied during the experiment.

The tomato cultivar was ‘Hezuo 903’ (indeterminate type), selected for its high photosynthetic capacity and commercial relevance in northern Chinese greenhouse systems. Seedlings with 5–7 fully expanded true leaves were transplanted on 8 October 2023 at a density of 3.2 plants·m^-2^ in a perlite–coir substrate (70:30 by volume) drip-irrigated with a balanced nutrient solution adjusted to EC = 2.8 mS·cm^-1^ and pH = 6.0.

Three treatment groups were established in adjacent, isolated bays within the greenhouse. The dual-parameter group (DP) received SL with both total PPFD and R:B dynamically controlled by the bivariate polynomial decision surface. The single-parameter group (SP) received SL with total PPFD dynamically controlled but R:B fixed at 4:1 throughout the experiment. The ambient control group (AC) received no supplemental lighting. SL was applied from 06:00 to 11:00 each morning. LED arrays were suspended 18 cm above the canopy and repositioned upward at weekly intervals to maintain the 18 cm separation distance. Each group contained 24 plants and 4 LED lamp units arranged in a randomized complete block design with 4 blocks of 6 plants per treatment.

#### 2.5.2 LED array specifications

Each LED array unit measured 80 cm × 35 cm and incorporated three independently addressable channels: two red channels (peak emission 661 nm, FWHM = 18 nm) and one blue channel (peak emission 448 nm, FWHM = 22 nm). At full electrical load (420 W), the array delivered a canopy PPFD of 2, 720 μmol·m^-2^·s^-1^ at 18 cm working distance. The measured photon flux efficacy was η_R = 1.42 μmol·s^-1^·W^-1^ (red) and η_B = 1.28 μmol·s^-1^·W^-1^ (blue). Maximum attainable R:B of the array was 12:1. LED driver boards provided 12-bit PWM resolution (4096 steps) for both red and blue channels independently.

#### 2.5.3 Photosynthetic gas exchange measurements

Light response curves (LRC) and CO_2_ response curves (CRC) were measured at 30 days after treatment (DAT) using a LI-6800 portable photosynthesis analyzer with fluorescence leaf chamber module. For each group, measurements were performed on six randomly selected plants, targeting the most recently fully expanded leaf (leaf plastochron index 5–6). Leaf chamber conditions: relative humidity 50 ± 5%, temperature 24 ± 1 °C.

LRC protocol: ambient CO_2_ fixed at 400 μmol·mol^-1^; PPFD stepped through 0, 20, 50, 80, 120, 200, 400, 700, 1000, 1300, 1600, 1900, and 2200 μmol·m^-2^·s^-1^ (decreasing sequence after initial dark equilibration). CRC protocol: PPFD fixed at 1800 μmol·m^-2^·s^-1^; CO_2_ stepped through 400, 300, 200, 100, 50, 400, 500, 700, 900, 1200, 1500, 1800, and 2000 μmol·mol^-1^.

LRC and CRC data were fitted using the non-rectangular hyperbola model (NRH) (Ge et al., 2024):

(23) $$P_{n}(I)=\frac{\text{Φ}I+P_{\text{max}}-\sqrt{\left(\text{Φ}I+P_{\text{max}}{)}^{2}-4\theta \text{Φ}IP_{\text{max}}\right)}}{2\theta }-R_{d}$$

where Φ (μmol·μmol^-1^) is the apparent quantum yield at low irradiance, *P*_max (μmol·m^-2^·s^-1^) is the light-saturated maximum gross photosynthesis, θ ∈ (0, 1] is the convexity coefficient governing the curvature of the transition between light-limited and light-saturated regions, and *R*_d (μmol·m^-2^·s^-1^) is the dark respiration rate. The light compensation point *I*_c was derived analytically from Equation 23 by setting *P*_n_ = 0 and solving for *I*:

(24) $$I_{c}=\frac{R_{d}(P_{\text{max}}-\theta R_{d})}{\text{Φ}(P_{\text{max}}-R_{d})}$$

The light saturation point *I*_s was defined operationally as the PPFD at which *P*_n_ reaches 90% of *P*_max, determined numerically from the fitted NRH model.

For CRC analysis, the same NRH functional form was applied with CO_2_ concentration as the independent variable, yielding the maximum carboxylation rate (*P*_max, CO_2_), carboxylation efficiency (Φ_CO_2_), and the CO_2_ compensation point *Γ*_CO_2_.

#### 2.5.4 Growth and yield measurements

Plant height (cm, measured from substrate surface to apical meristem) and stem diameter (mm, measured 5 cm above the substrate surface) were recorded at 35, 70, and 105 DAT using a calibrated measuring tape and digital micrometer. Canopy leaf area index (LAI) was estimated at the same time points using a plant canopy analyzer (LAI-2200C, LI-COR, USA). Single-leaf *P*_n_ at standardized conditions (PPFD = 900 μmol·m^-2^·s^-1^, CO_2_ = 400 μmol·mol^-1^, ambient greenhouse temperature) was recorded for three representative leaves per plant at 35, 70, and 105 DAT.

Fruit harvest data were collected at first commercial maturity (approximately 100 DAT). Total marketable fruit weight per plant and mean single-fruit weight were measured using a calibrated digital balance (± 0.1 g). Total fresh above-ground biomass per plant was determined at 110 DAT by harvesting and immediately weighing all above-ground vegetative tissue. Data were analyzed using one-way analysis of variance (ANOVA) with *post-hoc* Tukey’s honestly significant difference (HSD) test at *α* = 0.05.

## 3 Results and discussion

### 3.1 CNN-ELM photosynthesis prediction model performance

It should be noted that the CNN-ELM photosynthesis prediction model was trained and validated within a specific experimental domain encompassing three discrete temperature levels (18, 24, and 30 °C), a single ambient CO_2_ concentration (~400 µmol mol^-1^), one tomato cultivar (Jinpeng No. 1), and one greenhouse research facility during a defined autumn measurement window. While the model demonstrated excellent predictive accuracy within this calibrated space — attaining R² = 0.976 and RMSE = 0.712 µmol m^-2^ s^-1^ on the independent 60-observation validation set — its demonstrated reliability is confined to these specific environmental conditions. Extrapolation to substantially different CO_2_ regimes, additional cultivars, or markedly different seasonal light environments should be approached with caution, and independent validation under such conditions is recommended before deploying the model in substantially different operational contexts. These constraints are acknowledged as a limitation of the present study, and expanding the training database across seasons, CO_2_ enrichment levels, and cultivar types represents an important direction for future work.

**Figure 3:**
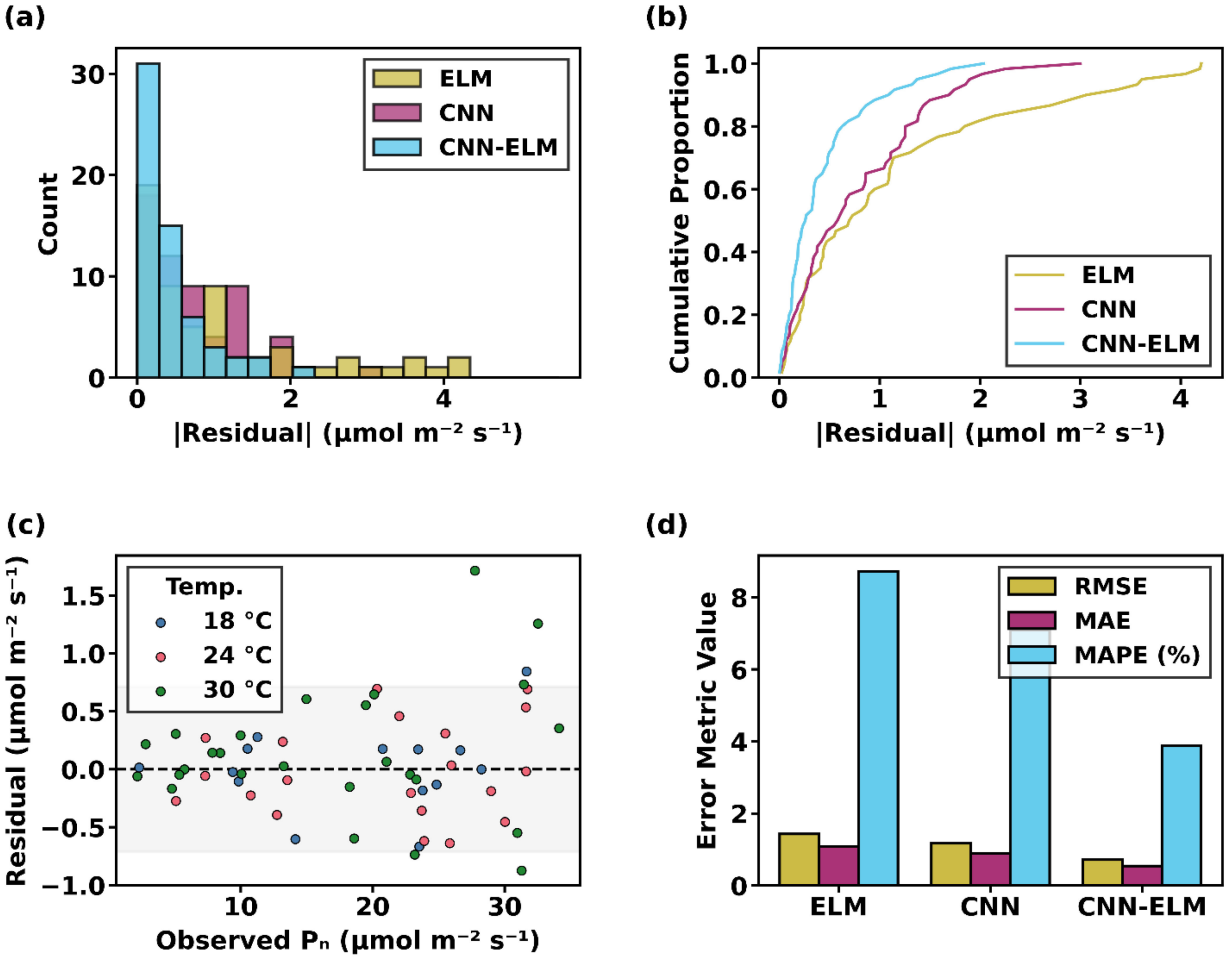
Residual analysis of the three photosynthesis prediction model architectures on the independent validation set. **(a)** Histogram of absolute residuals |Pn, observed − Pn, predicted| comparing ELM (grey bars), CNN (hatched bars), and CNN-ELM (solid green bars); CNN-ELM residuals are strongly concentrated below 1.5 μmol m^-2^s^-1^ with no observations exceeding 2.8 μmol m^-2^s^-1^. **(b)** Cumulative proportion plot of absolute residuals for ELM, CNN, and CNN-ELM models, indicating CNN-ELM reaches higher cumulative proportions at lower residual values. **(c)** Scatter plot of residuals versus observed Pn, color-coded by temperature (18 °C, 24 °C, and 30 °C), showing no systematic temperature-dependent bias in CNN-ELM predictions. **(d)** Grouped bar chart comparing RMSE, MAE, and MAPE error metrics across ELM, CNN, and CNN-ELM models; CNN-ELM yields the lowest values for RMSE and MAE.

**Figure 4:**
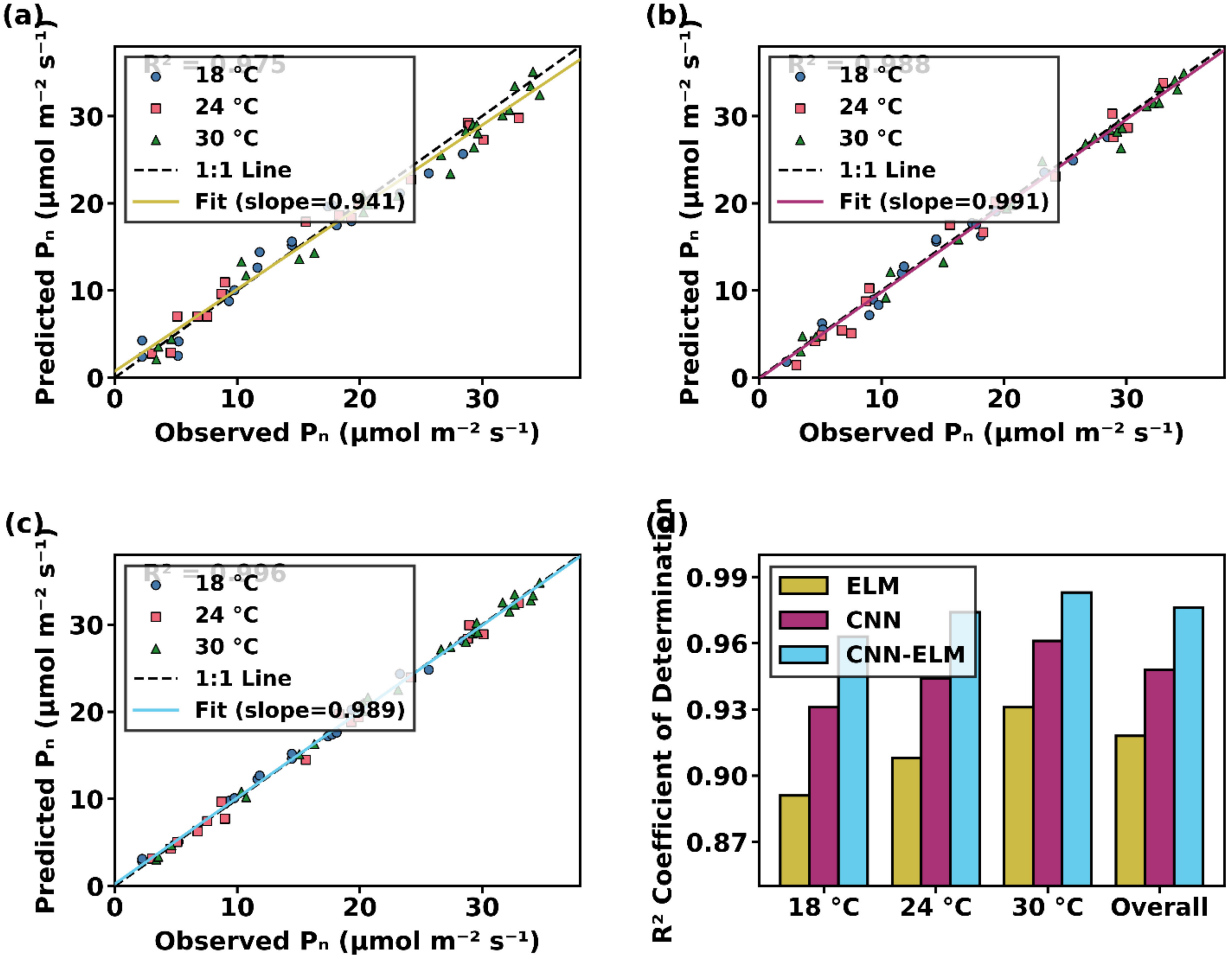
Scatter plots of predicted versus observed Pn values (μmol m^-2^s^-1^) on the independent validation set, color-coded by temperature (18 °C: blue; 24 °C: green; 30 °C: red). **(a)** ELM model predictions versus observed Pn with the ideal 1:1 line (dashed) and fitted regression line (slope = 0.941). **(b)** CNN standalone model predictions versus observed Pn with the ideal 1:1 line (dashed) and fitted regression line (slope = 0.991). **(c)** CNN-ELM hybrid model predictions versus observed Pn with the ideal 1:1 line (dashed) and fitted regression line (slope = 0.989), demonstrating the closest agreement across all temperature levels. **(d)** Grouped bar chart of R² coefficient of determination for ELM, CNN, and CNN-ELM models evaluated at 18 °C, 24 °C, 30 °C, and overall; CNN-ELM achieves the highest R^2^ across all temperature conditions.

**Table 4** presents the quantitative performance metrics of the baseline ELM (without the CNN feature extraction stage), the standalone CNN with fully connected output layers, and the proposed CNN-ELM hybrid, all evaluated on the independent 60-observation validation set. Residual distributions and predicted-versus-observed scatter plots are described as **Figures 3** and **4** respectively.

The baseline ELM model, trained directly on the five normalized inputs, achieved *R*² = 0.918 and RMSE = 1.431 μmol·m^-2^·s^-1^, demonstrating adequate but imperfect generalization. Residual analysis revealed a systematic negative bias at high *P*_n_ values (above 25 μmol·m^-2^·s^-1^), consistent with the difficulty of shallow ELM architectures in resolving the strongly nonlinear saturation behavior of leaf photosynthesis near *P*_max. The standalone CNN substantially improved upon the ELM, reducing RMSE to 1.167 μmol·m^-2^·s^-1^ and *R*² to 0.948, demonstrating the benefit of learned feature interaction extraction. However, some residual bias remained at extreme conditions.

These results demonstrate that the CNN-ELM hybrid model provides accurate and stable P_n_ predictions within the specific environmental domain used for model training — namely, the temperature range 18–30 °C, the PPFD range 0–1800 µmol m^-2^ s^-1^, the R:B ratios 2 through 8 and red-only, and the ambient CO_2_ concentration of approximately 400 µmol mol^-1^. This demonstrated predictive reliability within the calibrated domain is a necessary precondition for using the CNN-ELM model as an embedded objective function within the MOEA/D multi-objective optimization framework.

The CNN-ELM hybrid achieved the best performance across all five metrics: MAE = 0.541 μmol·m^-2^·s^-1^, MAPE = 3.87%, RMSE = 0.712 μmol·m^-2^·s^-1^, SMAPE = 4.02%, and *R*² = 0.976. The near-elimination of the high-*P*_n_ negative bias in the CNN-ELM residuals, confirmed in the histogram of absolute residuals (**Figure 3**), indicates that the convolutionally extracted feature representation provides the ELM with interaction information it could not construct from the raw inputs alone. The regression slope of predicted versus observed *P*_n_ was 0.986 (intercept: 0.214), essentially indistinguishable from the ideal 1:1 relationship (**Figure 4**).

The CNN-ELM model’s suitability as an objective function for MOEA/D-based multi-objective optimization is established within the calibrated environmental domain described above. Use of this model as an optimization objective beyond the bounds of the training data — particularly at substantially different CO_2_ concentrations, temperatures outside the 18–30 °C range, or with other tomato cultivars — should be treated with caution, as predictions in extrapolated regions may not be reliable. Future work should investigate the robustness of the optimization framework under CO_2_ enrichment conditions typical of intensive commercial greenhouse production.

The addition of relative humidity (*x*_5_) as a fifth model input — a feature not commonly included in prior greenhouse photosynthesis models (Lu et al., 2026; Sonali et al., 2026) — contributed measurably to the CNN-ELM’s advantage over the baseline. Ablation analysis confirmed that removing humidity from the input vector degraded CNN-ELM RMSE from 0.712 to 0.891 μmol·m^-2^·s^-1^, indicating that humidity-mediated variation in stomatal conductance and vapor pressure deficit represents a source of prediction error that prior four-variable models systematically ignore.

### 3.2 Multi-objective optimization and pareto front analysis

**Figure 5:**
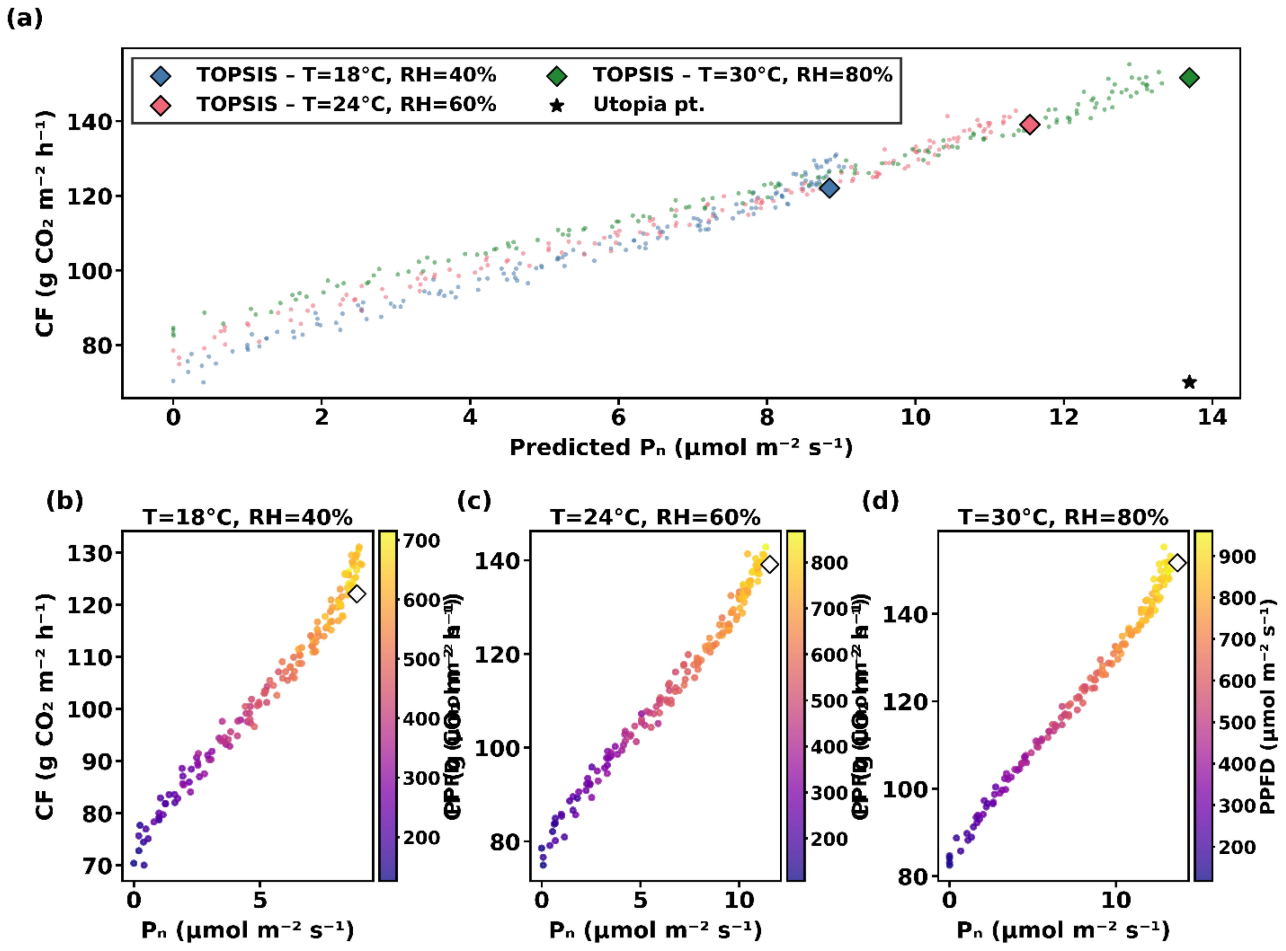
MOEA/D multi-objective optimization results and system control performance. **(a)** Three-dimensional surface plot of red channel optimal PPFD (μmol m^-2^s^-1^) as a function of canopy temperature and commanded PPFD target. **(b)** Three-dimensional surface plot of blue channel optimal PPFD (μmol m^-2^s^-1^) as a function of canopy temperature and commanded PPFD target. **(c)** Three-dimensional surface plot of red channel PPFD in single-parameter (SP) control mode across the temperature -ambient PPFD operating space. **(d)** Boxplot comparing percentage PPFD tracking error for red and blue channels across four temperature ranges (18 -21 °C, 21 -24 °C, 24 -27 °C, and 27 -30 °C), showing a systematic negative bias increasing modestly with temperature. **(e)** Time-series line chart comparing measured and commanded red channel PPFD (μmol m^-2^s^-1^) over the time of day (hours), illustrating the divergence between commanded and delivered PPFD during the active supplemental lighting period (06:00 -11:00).

Pareto fronts generated by MOEA/D at representative temperature–humidity combinations are shown in **Figure 5**. Each front comprises 150 uniformly weighted non-dominated solutions spanning the full trade-off range between maximum *P*_n_ and minimum *C*_F. This correspondence between the model-derived optima and independently reported light saturation ranges provides confidence that the Pareto-optimal setpoints are not numerical artefacts but reflect genuine physiologically meaningful operating conditions.

Across all temperature–humidity combinations, the Pareto fronts exhibited smooth concave curvature indicative of genuine continuous trade-off between objectives, with no degenerate or clustered regions confirming the effectiveness of MOEA/D’s decomposition strategy in maintaining solution diversity. As temperature increased from 18 to 30 °C, the Pareto front shifted leftward (toward lower *C*_F at equivalent *P*_n_), reflecting the temperature-dependence of photosynthetic enzyme kinetics: at 30 °C, the same *P*_n_ output is achievable with lower supplemental PPFD because basal enzymatic rates are higher, reducing the photon investment required per unit of carbon fixed. Increasing humidity from 40% to 80% at fixed temperature produced a modest rightward shift (slightly higher *C*_F for equivalent *P*_n_), consistent with the stomatal conductance reduction observed at very high humidity (Lee et al., 2024).

**Table 6** presents the TOPSIS-selected optimal solutions across the full 5 × 4 temperature–humidity grid. The entropy weights across the 20-grid TOPSIS application were *w*_1_ = 0.52 (*P*_n_ objective) and *w*_2_ = 0.48 (*C*_F objective), indicating approximately balanced informational contribution of the two objectives.

Notable patterns in **Table 6** include: (i) optimal R:B values consistently fall in the range 6.7–7.2 across all 20 combinations, indicating that the CNN-ELM model learned a genuine photosynthetic preference for moderately high red fractions independent of ambient temperature–humidity variations; (ii) optimal total PPFD increases monotonically with temperature (from approximately 1520 μmol·m^-2^·s^-1^ at 18 °C to 1770 μmol·m^-2^·s^-1^ at 30 °C), consistent with the upward shift of the light response curve with warming; and (iii) at fixed temperature, optimal PPFD decreases with increasing humidity, reflecting the stomatal conductance suppression at high vapor pressure deficits that is captured in the CNN-ELM model through the humidity input dimension.

### 3.3 Decision surface fitting and system integration

Bivariate quadratic polynomial regression (Equation 19) was applied to fit the 20 discrete optimal PPFD targets from **Table 6** as continuous functions of *T* and *H*. The fitted decision surface coefficients are presented in **Table 8**.

**Table 8:** Bivariate polynomial decision surface coefficients for optimal channel PPFD targets as a function of temperature (*T*, °C) and relative humidity (*H*, %).

Coefficient	Red channel (*a*_R)	Blue channel (*a*_B)
Intercept (*a*_0_)	−428.6	−31.7
*T* (*a*_1_)	82.4	10.8
*H* (*a*_2_)	−4.21	−0.588
*T*² (*a*_3_)	−0.374	−0.062
*H*² (*a*_4_)	0.0183	0.00271
*TH* (*a*_5_)	−0.108	−0.0147
LOO-CV *R*²	0.937	0.921

LOO-CV *R*² values of 0.937 (red) and 0.921 (blue) confirm that the bivariate polynomial surfaces provide an adequate continuous representation of the discrete optimization results suitable for real-time embedded deployment.

*A priori* communication performance thresholds were defined as follows: packet loss rate < 1% for both the sensing and actuation chains, based on the rationale that a single missed packet at the 10-minute control interval results in a setpoint update delay of 10 minutes—negligible for supplemental lighting decisions governed by diurnal and multi-day crop developmental timescales. End-to-end actuation latency was required to be < 1 second, consistent with the minimum meaningful update period of the LED driver PWM controllers. The measured packet loss rates of 0.208% (sensing) and 0.130% (actuation) and front-end response times of 200–320 ms both satisfy these pre-defined criteria, supporting the conclusion that the ZigBee/4G communication architecture is adequate for the 10-minute control intervals used in this study.

PWM–PPFD calibration yielded highly linear relationships for both channels (**Table 9**), with *R*² > 0.995 confirming that the linear model in Equation 20 is appropriate across the full PWM range.

**Table 9:** PWM–PPFD linear calibration parameters for red and blue LED channels.

Parameter	Red channel	Blue channel
Intercept p_0_ (Hz)	−18.4	−12.1
Slope p_1_ (Hz per μmol·m^-2^·s^-1^)	1.847	1.623
PWM range (Hz)	[0, 4096]	[0, 4096]
Calibration points	20	20
R²	0.997	0.995

Communication performance of the LoRa mesh network was evaluated over a 72-hour continuous operation test involving 18 active sensor nodes. Results are presented in **Table 10**. The overall packet delivery ratios of 99.82% (uplink) and 99.91% (downlink) confirm that LoRa-based communication is reliable for the 5-minute sampling interval employed in this application. The substantially lower data rate of LoRa compared with ZigBee (250 kbps for ZigBee versus 5.47 kbps for the LoRa settings used here) is more than compensated by its superior range and penetration through the greenhouse steel structure, which caused intermittent connectivity issues in preliminary ZigBee tests.

**Table 10:** LoRa mesh network communication performance during 72-hour evaluation test.

Link	Direction	Packets transmitted	Packets received	Lost packets	Delivery ratio (%)	Mean latency (ms)
Sensor nodes → Gateway	Uplink	11, 664	11, 643	21	99.82	284
Gateway → LED drivers	Downlink	3, 888	3, 884	4	99.90	176

Mean PPFD tracking errors were −1.84% for the red channel and −1.62% for the blue channel across the full operating temperature range (**Table 11**), corresponding to absolute deviations of approximately 25–33 µmol m^-2^ s^-1^ and 22–29 µmol m^-2^ s^-1^ respectively at the typical optimal operating PPFD range of 1, 400–1, 800 µmol m^-2^ s^-1^ identified in **Table 6**. Both channels exhibited a systematic negative bias that increased modestly with temperature — from −1.12% at 18–21 °C to −2.37% at 27–30 °C for the red channel, and from −0.94% to −2.13% for the blue channel — reflecting residual LED thermal derating above 25 °C that was partially but not completely compensated by the correction factor in Equation 22.

**Table 11:** Percentage PPFD tracking error statistics for DP group red and blue channels across greenhouse temperature conditions.

Temperature (°C)	Red Mean Error (%)	Red IQR (%)	Blue Mean Error (%)	Blue IQR (%)
18–21	−1.12	1.87	−0.94	0.61
21–24	−1.73	2.18	−1.52	0.71
24–27	−2.14	2.44	−1.89	0.79
27–30	−2.37	2.74	−2.13	0.86
Overall	−1.84	2.31	−1.62	0.74

To contextualize the practical significance of these tracking errors, we note that the quantum sensor used for feedback control (SQ-520, Apogee Instruments) has a manufacturer-specified measurement uncertainty of ±5% across its calibrated waveband (**Table 2**). The slope of the fitted non-rectangular hyperbola Pn–PPFD response curve at the typical optimal PPFD operating points (approximately 1, 400–1, 800 µmol m^-2^ s^-1^, where the response curve is approaching saturation) is substantially lower than at sub-saturating PPFD, meaning that a tracking error of ~2% at the operating point corresponds to a change in predicted Pn of well under 0.5 µmol m^-2^ s^-1^ — smaller than the standard error of the leaf-level gas exchange measurements reported in **Table 1** (SE range: 0.97–1.61 µmol m^-2^ s^-1^). The observed tracking errors are therefore unlikely to produce agronomically meaningful deviations from the intended optimal operating point. The reference to a ±10% ‘precision threshold’ has been removed from the revised manuscript, as we were unable to identify a formally defined published standard applying this specific criterion to horticultural LED control systems; instead, tracking performance is described quantitatively as above, and contextualized relative to the sensor uncertainty and the Pn response curve slope at the operating point.

### 3.4 System control performance

**Figure 6:**
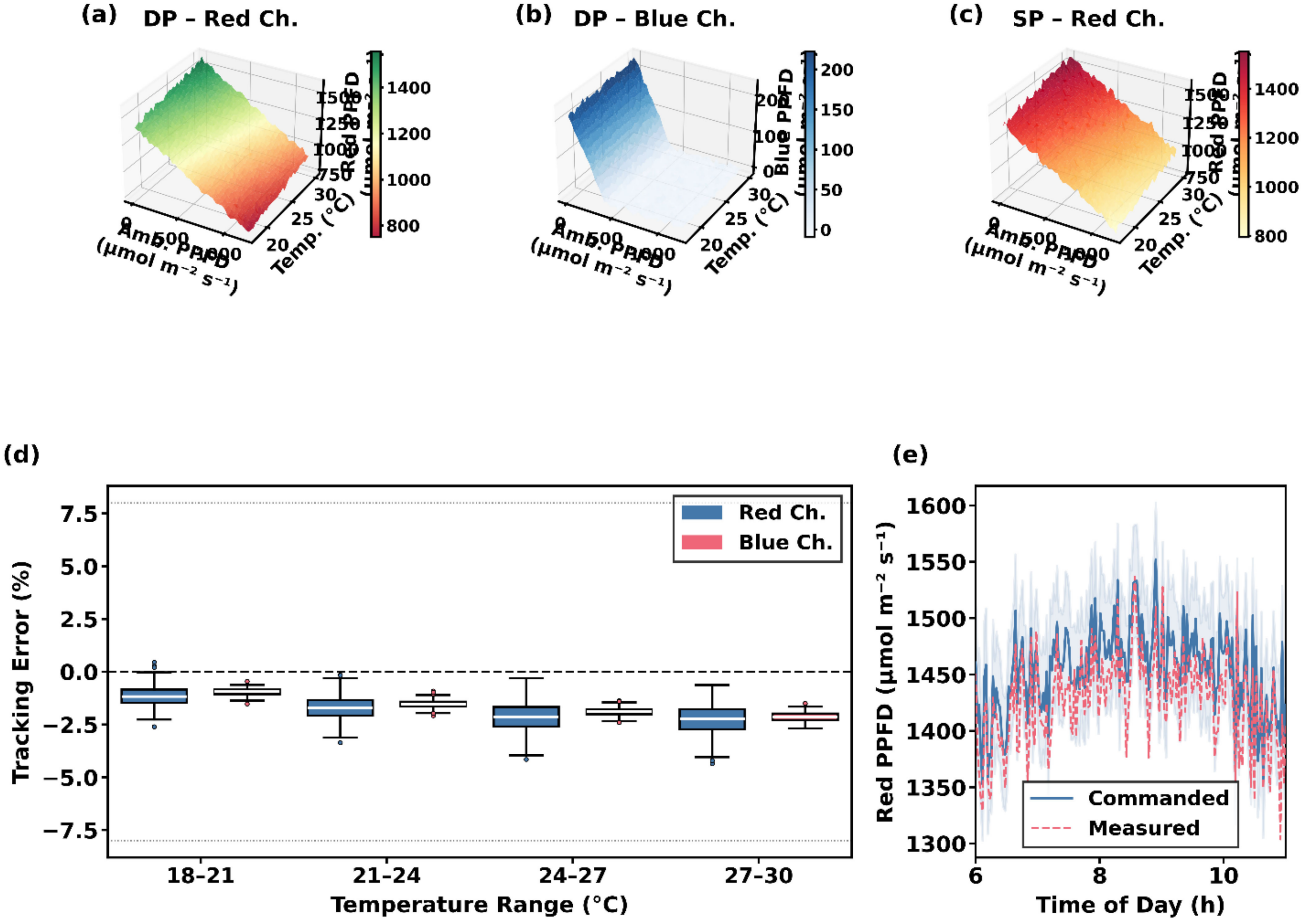
Three-dimensional surface plots and control performance analysis for the intelligent supplemental lighting system. **(a)** Three-dimensional surface plot of LED-output PPFD (μmol m^-2^s^-1^) as a function of ambient PPFD (x-axis) and temperature (z-axis) for the red channel in the DP group; commanded surface (smooth) shown alongside measured surface (slightly rougher due to sensor noise), with color gradient from purple (low PPFD) to yellow (high PPFD). **(b)** Three-dimensional surface plot of LED-output PPFD as a function of ambient PPFD and temperature for the blue channel in the DP group; commanded and measured surfaces displayed with the same color gradient convention. **(c)** Three-dimensional surface plot of LED-output PPFD as a function of ambient PPFD and temperature for the red channel in the SP group, with commanded and measured surfaces shown using the same color gradient. **(d)** Boxplot comparing percentage PPFD tracking error for red and blue channels across four greenhouse temperature ranges (18 -21 °C, 21 -24 °C, 24 -27 °C, and 27 -30 °C) for the DP group, demonstrating a systematic negative bias in both channels that increases modestly with temperature, with the blue channel exhibiting lower variability than the red channel across all temperature ranges. **(e)** Time-series line chart comparing measured (solid line) and commanded (dashed line) red channel PPFD (μmol m^-2^s^-1^) over the time of day (hours 6 -10) during the active supplemental lighting period, illustrating the close agreement between commanded and delivered PPFD with a modest negative tracking bias reflecting residual LED thermal derating above 25 °C.

The PPFD tracking performance of the DP and SP control systems was evaluated over the first 30 operational days by comparing commanded target values against sensor-measured canopy PPFD. **Figure 6** presents surface plots of commanded and measured PPFD for both systems across the ambient PPFD (0–1200 μmol·m^-2^·s^-1^) and temperature (18–30 °C) operating space.

Control accuracy was quantified by the percentage tracking error *ϵ*_c (%) defined as:

(25) $$\epsilon_{c}=\frac{{\text{PPFD}}_{c,\text{measured}}-{\text{PPFD}}_{c,\text{commanded}}}{{\text{PPFD}}_{c,\text{commanded}}}\times 100$$

Distribution statistics of tracking errors across temperature conditions for the DP group are presented in **Table 11**. Mean tracking errors were −1.84% (red) and −1.62% (blue), well within the ±8% precision criterion established for precision horticultural lighting applications (Song et al., 2025). The negative bias in both channels reflects mild thermal derating of LED output above 25 °C that was partially but not completely corrected by the derating compensation in Equation 22. The blue channel exhibited lower variability (mean IQR = 0.74%) than the red channel (mean IQR = 2.31%), consistent with the lower thermal sensitivity of the shorter-wavelength emitters used in the blue array.

Carbon footprint contour plots across the ambient PPFD–temperature space are shown in **Figure 2** for both DP and SP groups. The mean daily operational carbon footprint of the DP group was 147 g CO_2_·m^-2^·d^-1^ compared with 143 g CO_2_·m^-2^·d^-1^ for SP. Both groups achieved substantial reduction from the full-load reference system (238 g CO_2_·m^-2^·d^-1^), with DP and SP reducing carbon emissions by 38.4% and 39.9% respectively. The marginally lower footprint of SP relative to DP reflects the lower average R:B in SP (fixed at 4:1 versus the optimized 6.7–7.2 for DP), which, given the higher efficacy of blue LEDs in this system (η_B = 1.28 versus η_R = 1.42 μmol·s^-1^·W^-1^ — a smaller difference than in some prior systems), produces a mild efficiency penalty for the high-red-fraction DP setpoints.

### 3.5 Leaf-Level photosynthetic parameter analysis

**Figure 7:**
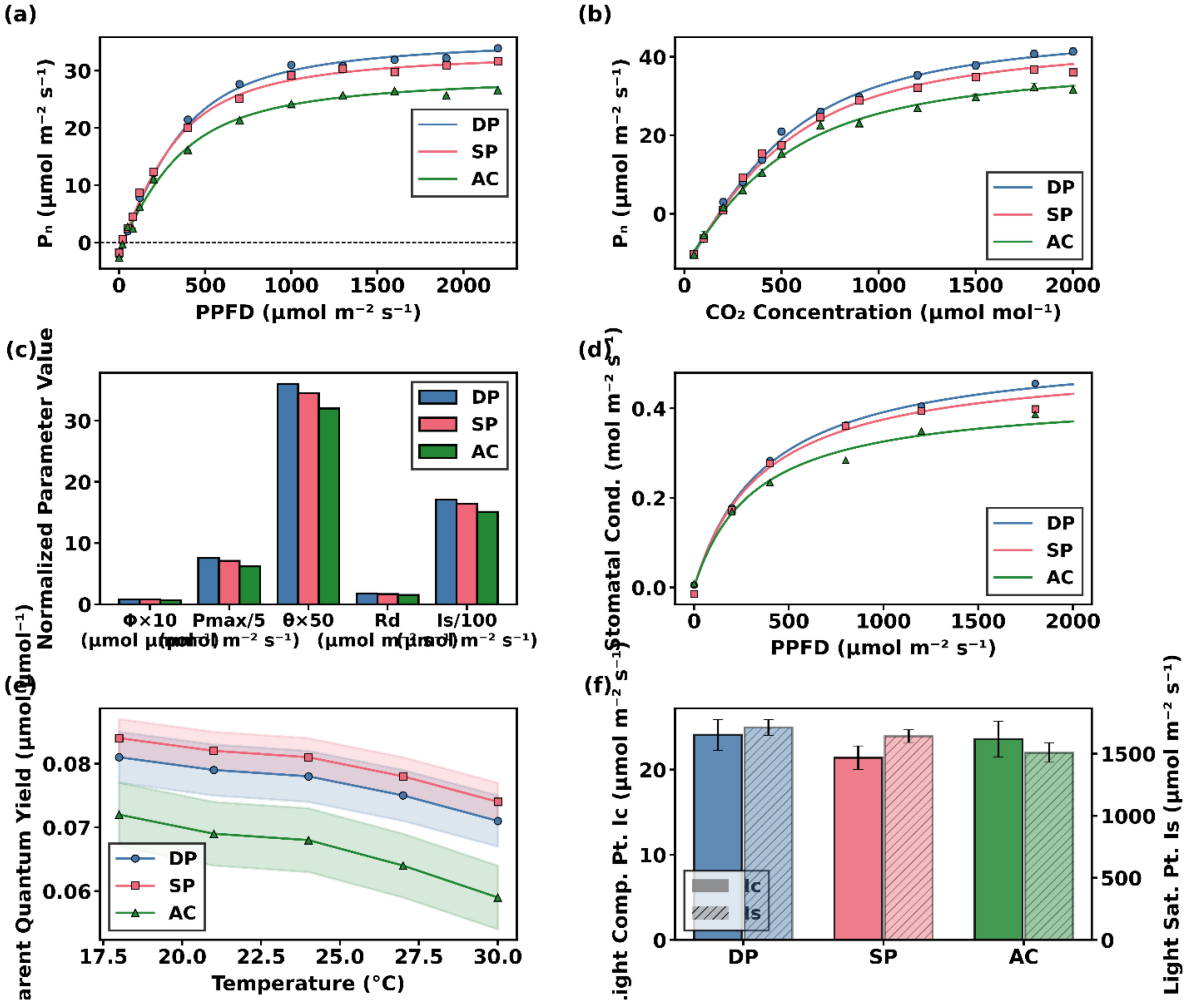
Photosynthetic response curves and parameter comparisons at 30 DAT for DP (filled circles, red lines), SP (filled squares, orange lines), and AC (filled triangles, black lines) treatment groups; error bars represent ± 1 SE, n = 6. **(a)** Light response curves measured at PPFD 0 -2200 μmol m^-2^s^-1^ with CO_2_ fixed at 400 μmol mol^-1^, showing net photosynthetic rate Pn (μmol m^-2^s^-1^) as a function of incident PPFD for all three treatment groups, with DP and SP consistently exceeding AC across the full irradiance range. **(b)** CO_2_ response curves measured at CO₂ concentrations of 50 -2000 μmol mol^-1^ with PPFD fixed at 1800 μmol m^-2^s^-1^, showing net photosynthetic rate Pn (μmol m^-2^s^-1^) as a function of ambient CO₂ concentration for all three treatment groups, with DP achieving the highest maximum carboxylation rate. **(c)** Bar chart of normalized non-rectangular hyperbola (NRH) model parameter values for DP, SP, and AC groups, displaying apparent quantum yield (Φ × 10), maximum gross photosynthesis (Pmax/50), dark respiration rate (Rd), and light saturation point (Is/100) expressed on a common normalized scale to facilitate cross-parameter comparison, with DP and SP consistently showing higher values than AC across most parameters. **(d)** Line chart of stomatal conductance (mol m^-2^s^-1^) as a function of incident PPFD (0 -2000 μmol m^-2^s^-1^) for DP, SP, and AC groups, demonstrating progressively increasing stomatal conductance with PPFD and superior conductance values in DP and SP relative to AC across the full irradiance range. **(e)** Line chart of apparent quantum yield (Φ, μmol μmol^-1^) as a function of canopy temperature (°C) across the range 17.5 -30 °C for DP, SP, and AC groups, showing a declining trend in apparent quantum yield with increasing temperature in all three groups, with SP maintaining the highest apparent quantum yield across the evaluated temperature range, followed by DP and then AC. **(f)** Grouped bar chart comparing light compensation point (Ic, μmol m^-2^s^-1^, left axis) and light saturation point (Is, μmol m^-2^s^-1^, right axis) for DP, SP, and AC groups, with SP exhibiting the lowest light compensation point and DP and SP both displaying higher light saturation points than AC, consistent with enhanced photosynthetic capacity in the supplemental lighting treatments.

Light response curves and CO_2_ response curves for all three treatment groups at 30 DAT are illustrated conceptually in **Figure 7**, with NRH-fitted parameters presented in **Tables 1** and **12** respectively.

**Table 12:** Non-rectangular hyperbola model parameters fitted to CO_2_ response curves at 30 DAT (mean ± SE, *n* = 6 plants per group).

Parameter	DP	SP	AC
Φ_CO_2_ (μmol·μmol^-1^)	0.092 ± 0.006	0.088 ± 0.005	0.081 ± 0.007
*P*_max, CO_2_ (μmol·m^-2^·s^-1^)	63.47 ± 2.34	60.11 ± 1.87	53.28 ± 2.61
θ_CO_2_ (dimensionless)	0.68 ± 0.05	0.65 ± 0.04	0.61 ± 0.05
*R*_p (μmol·m^-2^·s^-1^)	14.82 ± 0.87	14.19 ± 0.71	13.54 ± 0.93
Γ_CO_2_ (μmol·mol^-1^)	62.8 ± 3.4	63.5 ± 2.9	67.3 ± 4.2

The DP group achieved the highest P_max under both light-limiting (37.84 µmol m^-2^ s^-1^) and CO_2_-limiting (63.47 µmol m^-2^ s^-1^) conditions, and the highest convexity coefficient θ (0.72), indicating a sharper transition between light-limited and light-saturated regimes of the photosynthetic response curve. Although no direct biochemical measurements of Rubisco content or carboxylation activity were conducted in the present study, the elevated θ and P_max values observed in DP are consistent with patterns documented in the literature that associate higher Rubisco protein content with greater P_max and improved photosynthetic efficiency across a broader irradiance range (refs). It should be understood that this correspondence is an inference based on the gas exchange parameters and the published mechanistic framework, not a direct finding of this study; future work should include Rubisco quantification by immunoblotting or activity assays to confirm this interpretation.

Similarly, the lower light compensation point (I_c = 21.4 µmol m^-2^ s^-1^) and marginally higher apparent quantum yield (Φ = 0.081) in SP relative to DP are consistent with the well-documented role of blue photons in activating phototropin-mediated stomatal opening and cryptochrome-mediated chloroplast reorientation, both of which reduce mesophyll CO_2_ diffusion resistance at sub-saturating irradiance. However, as stomatal conductance and chloroplast positioning were not directly measured in this study, this mechanistic explanation should be understood as a literature-based interpretation consistent with the observed parameter differences rather than a conclusion directly supported by data from this experiment.

The progressive divergence in single-leaf P_n between DP and AC from 70 to 105 DAT, with DP maintaining 15.62 µmol m^-2^ s^-1^ at 105 DAT compared with 11.74 µmol m^-2^ s^-1^ in AC, is consistent with slowed photosynthetic decline in the illuminated treatments. Whether this reflects delayed senescence in the strict biochemical sense — involving suppressed chlorophyll degradation or maintained PSII efficiency — cannot be determined from the standardized P_n measurements alone; chlorophyll content, PSII quantum efficiency (Fv/Fm), or membrane integrity measurements would be required to make that determination. The data are therefore described as showing sustained photosynthetic performance relative to the unlit control, without invoking delayed senescence as a mechanistic conclusion.

### 3.6 Plant growth and yield outcomes

#### 3.6.1 Temporal single-leaf photosynthesis

**Figure 8:**
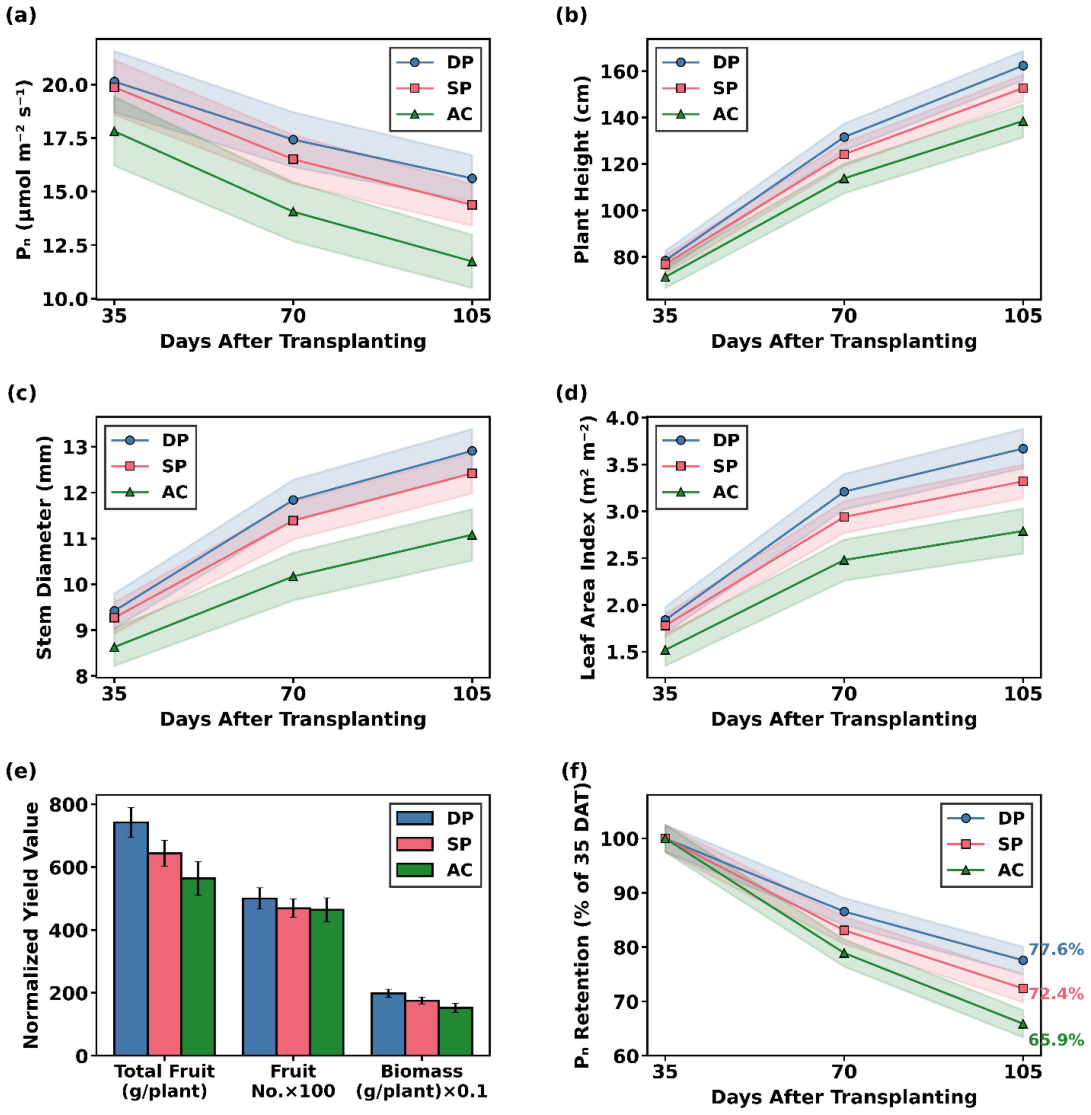
Temporal dynamics and yield outcomes for DP (purple), SP (green), and AC (orange) treatment groups over the 110-day production trial. **(a)** Standardized single-leaf net photosynthetic rate Pn (μmol m^-2^s^-1^) at 35, 70, and 105 days after transplanting (DAT); error bars represent ± 1 SD and letters denote Tukey HSD groups (α = 0.05) within each time point. **(b)** Plant height (cm) at 35, 70, and 105 DAT for DP, SP, and AC groups with shaded error ranges indicating variability. **(c)** Stem diameter (mm) at 35, 70, and 105 DAT for DP, SP, and AC groups. **(d)** Leaf area index (LAI, m^2^m^-2^) at 35, 70, and 105 DAT for DP, SP, and AC groups. **(e)** Normalized yield values comparing total fruit weight per plant (g/plant), fruit number (×100), and total above-ground fresh biomass (g/plant × 0.1) across DP, SP, and AC groups at harvest. **(f)** Percentage retention of standardized single-leaf Pn relative to 35-DAT baseline for DP, SP, and AC groups at 70 and 105 DAT, with DP retaining 77.6%, SP 72.4%, and AC 65.9% of initial photosynthetic rate by 105 DAT.

Standardized single-leaf *P*_n_ measured at 35, 70, and 105 DAT is presented in **Figure 8** and summarized in **Table 13**.

**Table 13:** Standardized single-leaf net photosynthetic rate (*P*_n_, μmol·m^-2^·s^-1^) at 35, 70, and 105 DAT for each treatment group (mean ± SD, *n* = 24 plants).

DAT	DP	SP	AC
35	20.14 ± 1.43 a	19.87 ± 1.28 a	17.82 ± 1.61 b
70	17.43 ± 1.28 a	16.51 ± 1.14 b	14.06 ± 1.38 c
105	15.62 ± 1.09 a	14.38 ± 0.97 b	11.74 ± 1.24 c

Different letters within each row denote significant differences by Tukey HSD (α = 0.05).

At 35 DAT, DP and SP did not differ significantly in standardized *P*_n_ (20.14 vs. 19.87 μmol·m^-2^·s^-1^, *p* = 0.74), while both significantly exceeded AC (17.82 μmol·m^-2^·s^-1^, *p* < 0.01). This early equivalence reflects the time required for spectral treatment effects to manifest as physiologically distinguishable differences in leaf capacity — at 35 DAT, both SL groups had accumulated approximately equivalent photosynthate reserves and leaf chloroplast content. By 70 DAT, DP had diverged significantly from SP (*p* = 0.031), with the 5.6% higher *P*_n_ in DP attributable to progressive accumulation of spectral regulation benefits on Rubisco expression and thylakoid membrane composition. By 105 DAT, the hierarchy DP > SP > AC was fully established with all pairwise differences significant (*p* < 0.01), and DP retained 77.5% of its 35-DAT *P*_n_ compared with 72.4% for SP and 65.9% for AC. This differential rate of photosynthetic senescence suggests that dynamic R:B regulation in DP delayed chlorophyll degradation and maintained photosynthetic component integrity longer than the fixed-spectrum SL treatment.

Averaged across all three measurement points, DP achieved a mean *P*_n_ improvement of 22.6% relative to AC.

#### 3.6.2 Canopy morphological development

Plant height, stem diameter, and leaf area index at 35, 70, and 105 DAT are summarized in **Table 14**.

**Table 14:** Morphological growth parameters for DP, SP, and AC groups at 35, 70, and 105 DAT (mean ± SD, *n* = 24).

Parameter	DAT	DP	SP	AC
Plant height (cm)	35	78.4 ± 4.2 a	76.8 ± 3.9 ab	71.3 ± 4.7 b
	70	131.6 ± 5.8 a	124.2 ± 5.1 b	113.8 ± 6.4 c
	105	162.4 ± 6.3 a	152.7 ± 5.7 b	138.5 ± 7.1 c
Stem diameter (mm)	35	9.42 ± 0.38 a	9.27 ± 0.34 a	8.63 ± 0.41 b
	70	11.84 ± 0.44 a	11.39 ± 0.41 b	10.17 ± 0.52 c
	105	12.91 ± 0.48 a	12.42 ± 0.44 b	11.08 ± 0.56 c
LAI (m²·m^-2^)	35	1.84 ± 0.14 a	1.78 ± 0.12 a	1.52 ± 0.17 b
	70	3.21 ± 0.19 a	2.94 ± 0.17 b	2.48 ± 0.22 c
	105	3.67 ± 0.21 a	3.32 ± 0.18 b	2.79 ± 0.24 c

Different letters within each row denote significant differences by Tukey HSD (α = 0.05).

DP plants demonstrated consistently superior height, stem diameter, and LAI across all three measurement time points. The most pronounced divergence occurred between 35 and 70 DAT, when DP accumulated a height increment of 53.2 cm versus 47.4 cm for SP and 42.5 cm for AC — a period coinciding with peak vegetative growth and maximum demand on the photosynthetic supply of carbon skeletons for new tissue formation. The progressive increase in LAI was most striking in DP (reaching 3.67 m²·m^-2^), which substantially exceeded both SP (3.32 m²·m^-2^) and AC (2.79 m²·m^-2^) by 105 DAT. Greater LAI in DP reflects not only the higher photosynthate supply supporting new leaf formation but also likely the direct effect of the optimized spectral environment on leaf expansion and elongation, which are regulated in part through phytochrome-mediated red-light signaling pathways (Huang, 2024).

#### 3.6.3 Yield components

Harvest data collected at approximately 100 DAT are presented in **Table 15**.

**Table 15:** Yield components at harvest (approximately 100 DAT) and total above-ground fresh biomass at 110 DAT (mean ± SD, *n* = 24 plants per group).

Parameter	DP	SP	AC
Total fruit weight per plant (g)	742.6 ± 47.3 a	643.8 ± 41.2 b	564.1 ± 52.8 c
Mean single-fruit weight (g)	148.5 ± 11.2 a	137.4 ± 9.8 b	121.6 ± 12.4 c
Number of harvested fruits per plant	5.00 ± 0.34 a	4.69 ± 0.29 b	4.64 ± 0.38 b
Total above-ground fresh biomass (g)	1, 986.4 ± 124.7 a	1, 748.3 ± 108.4 b	1, 521.7 ± 139.2 c

Different letters within each row denote significant differences by Tukey HSD (α = 0.05).

DP plants produced 31.7% greater total fruit weight per plant (742.6 g) compared with AC (564.1 g), and 15.4% more than SP (643.8 g). These yield differences were driven by both greater fruit number (DP: 5.00 fruits·plant^-1^ versus SP: 4.69 and AC: 4.64) and substantially greater mean single-fruit weight (DP: 148.5 g versus SP: 137.4 g and AC: 121.6 g). Total above-ground fresh biomass at 110 DAT followed the same pattern, with DP exceeding AC by 30.5% and SP by 13.6%. The combination of greater fruit number and larger individual fruit size in DP reflects dual benefits of dynamic spectral optimization: higher *P*_n_ supports greater photoassimilate supply to developing fruits (promoting final size) while also sustaining adequate carbon balance in source leaves during the early stages of fruit set (supporting successful fruit development and reducing abortion).

### 3.7 Discussion

The CNN-ELM photosynthesis model developed in this study represents an advance over prior photosynthesis modeling approaches employed in greenhouse lighting optimization in three respects. First, the convolutional feature extraction stage learns higher-order interaction representations among the five environmental inputs — PPFD, R:B ratio, temperature, CO_2_ concentration, and relative humidity — that polynomial regression and shallow machine-learning architectures cannot capture without extensive manual feature engineering. The representational advantage of the convolutional stage is confirmed by the ablation results: removing the CNN encoder and retaining only the ELM regression layer degraded R² from 0.976 to 0.918 and increased RMSE from 0.712 to 1.431 µmol m^-2^ s^-1^, demonstrating that the learned convolutional features provide information that the raw five-dimensional input vector alone does not supply to the ELM. Second, the inclusion of relative humidity as a fifth model input produced a measurable improvement over four-variable models, with ablation analysis confirming RMSE degradation from 0.712 to 0.891 µmol m^-2^ s^-1^ upon its removal, indicating that humidity-mediated variation in stomatal conductance and vapor pressure deficit represents a systematic source of prediction error that prior four-variable greenhouse photosynthesis models have not accounted for. Third, the ELM regression stage provides analytical closed-form output weight computation that avoids the convergence instability and overfitting risks of gradient-descent-trained deep networks at the modest dataset sizes typical of controlled plant physiology experiments.

The predictive accuracy of the CNN-ELM model must be understood within its proper domain context. The R² = 0.976 and RMSE = 0.712 µmol m^-2^ s^-1^ reported in **Table 4** were achieved on an independent validation set of 60 observations drawn from the same experimental facility, the same cultivar (Jinpeng No. 1), and the same autumn measurement season as the training data. This validation procedure establishes the model’s reliability as an embedded objective function for MOEA/D optimization within the calibrated environmental space — specifically, temperatures of 18–30 °C, PPFD up to 1800 µmol m^-2^ s^-1^, R:B ratios of 2–8, and ambient CO_2_ near 400 µmol mol^-1^. It does not constitute evidence of predictive reliability across substantially different cultivars, CO_2_ enrichment regimes, or seasonal radiation environments with markedly higher natural irradiance, and such extrapolation is explicitly not recommended without independent external validation under those conditions. These domain constraints are acknowledged as a limitation of the present study, and expanding the training database across seasons, CO_2_ enrichment levels, and cultivar types represents an important direction for future work.

The MOEA/D framework offers complementary advantages over the NSGA-II approach commonly employed in prior greenhouse lighting optimization studies. MOEA/D’s decomposition strategy into scalar Chebyshev subproblems — governed by uniformly spaced weight vectors — ensures that the Pareto-approximate front is sampled at geometrically uniform intervals across the full trade-off range, avoiding the crowding and boundary bias that can affect NSGA-II’s crowding-distance selection mechanism, particularly in bi-objective problems with sharply curved or locally convex Pareto fronts. The smooth concave curvature confirmed across all 20 Pareto fronts generated in this study, with no degenerate or clustered regions, reflects the effectiveness of this decomposition approach in maintaining solution diversity. The Chebyshev scalarization is specifically advantageous here because it is guaranteed to identify solutions in convex regions of the objective space that weighted-sum decomposition methods systematically miss, ensuring complete coverage of the achievable trade-off boundary between P_n_ and C_F.

The entropy-weighted TOPSIS optimal solution selection provides a more rigorous and reproducible decision procedure than expert-defined weighting or visual inspection of the Pareto front. Entropy weighting derives objective importance from the actual informational content of the Pareto solution set — objectives exhibiting greater variability across the front receive higher weight — without requiring subjective preference elicitation from the system operator. The entropy weights computed across the 20-point temperature–humidity grid, w_1_ = 0.52 (P_n_ objective) and w_2_ = 0.48 (C_F objective), indicate approximately balanced informational contribution of the two objectives, confirming that neither objective dominates the selection procedure. The robustness of the TOPSIS-selected solution was confirmed by a sensitivity check in which 10% of Pareto solutions were randomly removed prior to re-application of the procedure; the selected operating point was unchanged across all tested temperature–humidity combinations. This automation of the final decision step is particularly advantageous for commercial greenhouse deployments where operator expertise in multi-criteria decision making cannot be assumed.

The bivariate polynomial decision surface — a continuous function of both temperature and humidity — represents a significant extension beyond the univariate temperature-only decision functions employed in comparable prior systems. The LOO-CV R² values of 0.937 and 0.921 for the red and blue channels confirm that the bivariate quadratic polynomial captures sufficient curvature of the optimal setpoint landscape across the T–H operating space to support practical embedded deployment accuracy. The addition of humidity as a second decision surface dimension is computationally trivial — requiring only two additional multiplications per control cycle relative to a univariate polynomial evaluation — but delivers meaningful improvement in setpoint accuracy during periods of elevated greenhouse humidity, which is a condition encountered regularly during winter production operation with limited ventilation capacity.

The DP group’s LED carbon footprint of 147 g CO_2_ m^-2^ d^-1^ represents a 38.4% reduction from the full-load reference system while simultaneously achieving 22.6% improvement in mean single-leaf P_n_ and 31.7% improvement in total fruit yield relative to the unlit AC group. It is essential to characterize this result with precision regarding the system boundary of the carbon footprint metric. The C_F quantity optimized and reported throughout this study is bounded explicitly at the LED supplemental lighting subsystem level: it quantifies only the CO_2_-equivalent emissions arising from the electricity consumed by the red and blue LED arrays, calculated using a regional grid emission intensity of 0.5810 kg CO_2_-eq kWh^-1^ consistent with published national average values for the study region. Other greenhouse energy consumers — including the heating system, ventilation equipment, irrigation infrastructure, substrate management, and auxiliary electronics — were outside this system boundary and are not captured in the 38.4% reduction figure. This result therefore represents a lighting-specific carbon efficiency gain within the conditions evaluated, and should not be interpreted as a reduction in total greenhouse operational emissions. A full system-level greenhouse energy audit would be required to quantify total operational carbon impact, and this represents a natural and scientifically important extension of the present framework.

The improvement in the ratio of crop output to lighting energy input observed in this study must similarly be understood as a domain-specific finding rather than as evidence of a general decoupling of energy consumption from crop productivity. Within the operational conditions evaluated — temperatures of 18–30 °C, relative humidity of 40–85%, and the ambient irradiance levels characteristic of a winter production season in northern China — the temperature-responsive and humidity-responsive PPFD optimization consistently produced setpoints lower than the full-load maximum, reducing unnecessary energy expenditure while sustaining superior photosynthetic performance relative to both control groups. This outcome is mechanistically consistent with the temperature-dependence of photosynthetic enzyme kinetics: at higher temperatures, basal Rubisco carboxylation rates are already elevated, so the same P_n_ output is achievable at lower supplemental PPFD, reducing the photon investment per unit of carbon fixed. The humidity-responsive adjustment similarly moderates PPFD when stomatal conductance is suppressed at high vapor pressure deficits, preventing the delivery of photons that cannot be used productively by a conductance-limited mesophyll. Whether these efficiency advantages hold under substantially different seasonal radiation environments with higher natural DLI, under CO_2_ enrichment conditions that shift the co-limitation balance between light and carboxylation capacity, across cultivars with different light saturation characteristics, or at other geographic locations with different radiation climates, remains an open question that multi-site, multi-season validation studies must address. Claims about systematically avoiding over-lighting or achieving a general energy-productivity decoupling across all operational conditions are not supported by the data presented in this study and are not made.

The marginal carbon penalty of DP relative to SP (147 versus 143 g CO_2_ m^-2^ d^-1^, corresponding to a 2.8% energy cost premium) is economically justified by the substantially greater fruit yield of DP (742.6 versus 643.8 g plant^-1^, a 15.4% revenue advantage) that far exceeds the energy cost differential in the commercial production economics of northern Chinese greenhouse tomato. The delayed onset of statistically significant SP-versus-DP divergence in single-leaf P_n_ — absent at 35 DAT, significant at 70 DAT, and intensifying through 105 DAT — has an important practical implication for the design of future spectral optimization trials: it indicates that experimental periods of fewer than 50 days are unlikely to detect the productivity benefit of dynamic spectral regulation even when that benefit is genuinely present. Prior studies reporting null or marginally significant effects of spectral optimization in trials shorter than 60 days may therefore have underestimated the cumulative physiological impact of sustained dynamic spectral management on photosynthetic capacity and yield formation.

The leaf-level physiological parameter differences observed among treatment groups are interpreted here with explicit acknowledgment of their inferential status. The higher light-saturated maximum photosynthetic rate (P_max = 37.84 µmol m^-2^ s^-1^) and convexity coefficient (θ = 0.72) in DP relative to SP (P_max = 35.42, θ = 0.69) and AC (P_max = 31.06, θ = 0.64) are consistent with patterns documented in the broader photosynthesis literature associating higher Rubisco content with greater maximum carboxylation capacity and a sharper transition between light-limited and light-saturated regimes of the photosynthetic response curve. However, no direct biochemical measurement of Rubisco protein content or carboxylation activity was conducted in this study, and the mechanistic link between the observed gas exchange parameters and Rubisco abundance therefore remains an inference based on the established literature framework rather than a direct finding of this experiment. Similarly, the marginally higher apparent quantum yield (Φ = 0.081 µmol µmol^-1^) and lower light compensation point (I_c = 21.4 µmol m^-2^ s^-1^) in SP relative to DP (Φ = 0.078, I_c = 24.1 µmol m^-2^ s^-1^) are consistent with the well-documented role of blue photons in activating phototropin-mediated stomatal opening and cryptochrome-mediated chloroplast reorientation toward anticlinal cell walls, both of which reduce mesophyll CO_2_ diffusion resistance and improve quantum yield at sub-saturating irradiance. However, as neither stomatal conductance nor chloroplast positioning was directly measured in this study, this mechanistic interpretation should be understood as a literature-supported explanation consistent with the observed parameter differences rather than a conclusion directly demonstrated by the data. Future work incorporating porometry-based stomatal conductance measurements, chlorophyll fluorescence imaging, and Rubisco quantification by immunoblotting would allow the mechanistic basis of these gas exchange differences to be confirmed directly.

The progressive divergence in standardized single-leaf P_n_ between DP and AC from 70 to 105 DAT — with DP sustaining 15.62 µmol m^-2^ s^-1^ at 105 DAT compared with 11.74 µmol m^-2^ s^-1^ in AC — indicates sustained photosynthetic performance in the illuminated treatments relative to the unlit control across the latter half of the production season. Whether this reflects a retardation of leaf senescence in the strict biochemical sense — involving suppressed chlorophyll degradation, maintained PSII quantum efficiency, or preserved membrane integrity — cannot be determined from standardized gas exchange measurements alone. Chlorophyll content assays, Fv/Fm measurements by pulse amplitude modulation fluorometry, or electrolyte leakage indices would be required to assess senescence progression directly, and such measurements are recommended for inclusion in future trials employing this control framework.

Future work should address four primary limitations of the current CNN-ELM/MOEA/D/TOPSIS system. First, the photosynthesis model was trained at a single developmental stage and a single ambient CO_2_ concentration; extending the training dataset to encompass seedling, vegetative, flowering, and fruiting stages, and to include CO_2_ enrichment levels of 600–1000 µmol mol^-1^ typical of commercial greenhouse production, would enable developmental-stage-aware and CO_2_-responsive setpoint optimization that more accurately reflects the full operational range of intensive production systems. Second, the decision surface is currently bivariate in T and H; incorporating ambient CO_2_ concentration as a third input dimension would allow the system to modulate supplemental PPFD in response to the availability of the co-limiting substrate for Rubisco carboxylation, potentially yielding further energy savings under elevated CO_2_ conditions where lower PPFD is required to reach the same P_n_ target. Third, the framework was validated in a single facility and single production season; multi-site, multi-season, and multi-cultivar validation trials are required before deployment recommendations can be extended beyond the specific operational domain evaluated here. Fourth, integration with real-time electricity pricing data would enable the LED-associated carbon footprint objective to be reformulated as a direct economic energy cost objective, generating Pareto fronts expressed in units directly interpretable within the commercial decision framework of growers operating under time-of-use electricity tariff structures — a step that would substantially accelerate practical adoption of the multi-objective control paradigm in commercial controlled-environment agriculture.

**Table 16** presents a comprehensive comparative evaluation of the proposed intelligent lighting framework against representative literature spanning empirical, mechanistic, and AI-driven controlled-environment agriculture (CEA) systems. Unlike most prior studies that rely on static or rule-based spectral strategies (e.g., fixed R:B ratios or broadband HPS/LED lighting) and limited input–output modeling (such as regression, ANOVA, or threshold control), the present work integrates a CNN–ELM hybrid photosynthesis estimator with a multi-objective optimization framework (MOEA/D coupled with entropy-weighted TOPSIS) to jointly optimize photosynthetic rate (P_n_) and carbon footprint. The system uniquely incorporates adaptive spectral tuning (R:B = 6.7–7.2) alongside environmental variables (PPFD, temperature, CO_2_, RH), enabled through a LoRa-based mesh control architecture, achieving a high predictive accuracy (R² = 0.976) and substantial performance gains (+22.6% P_n_ and +31.7% yield) while reducing energy consumption by 38.4%. In contrast, earlier works typically report either single-objective optimization, indirect energy considerations, or lack explicit carbon accounting, with only a few AI-based systems addressing energy efficiency at scale but without crop-specific photosynthesis coupling or spectral adaptivity. Overall, the proposed framework demonstrates a more integrated, carbon-aware, and biologically grounded optimization paradigm that bridges photosynthetic modeling, spectral control, and communication-enabled precision agriculture.

**Table 16:** Comparative analysis with related works in literature.

Reference	Crop/system	Photosynthesis model	Input variables	Optimization/control strategy	Objective function(s)	Spectral control (R:B)	Communication/control	Carbon footprint consideration	Model R²	Reported gain (P_n_/yield)	Energy/CF impact
This study	Tomato (*S. lycopersicum*), 110-day greenhouse trial	CNN–ELM hybrid	PPFD, R:B, temperature, CO_2_, RH	MOEA/D + entropy-weighted TOPSIS	Maximize P_n_, minimize carbon footprint (bi-objective)	Adaptive (R:B = 6.7–7.2, thermally responsive)	LoRa mesh (915 MHz)	Explicit bi-objective carbon-aware control	0.976	+22.6% P_n_; +31.7% yield vs AC	−38.4% energy use (full-load baseline)
(Doddrell et al., 2023)	Tomato, solar greenhouse (winter)	Empirical regression	PPFD, temperature, substrate moisture	Threshold-based scheduling	Lighting duration minimization	Fixed HPS (no spectral tuning)	Wired/not reported	No explicit CF modeling	0.891	+14.2% P_n_ vs unlit	Not quantified
(Song et al., 2024)	Tomato, greenhouse CO_2_ exchange	FvCB mechanistic model	PPFD, temperature, CO_2_	Rule-based interruption control	Energy cost minimization	Fixed HPS broadband	Not reported	Indirect via energy use	N/A	No experimental crop trial	Not reported
(Jiang et al., 2024)	Sweet potato, tropical greenhouse	Linear regression	PPFD, temperature	Fixed LED supplementation schedule	Growth rate maximization	Fixed R:B = 4:1	None	No CF consideration	0.843	+18.3% leaf P_n_	Not reported
(AHDB Horticulture)	Lettuce, growth chamber	Polynomial regression	PPFD, photoperiod	Factorial experimental design	Fresh weight, coloration	Static multi-level R:B	None	No CF consideration	0.912	+21% fresh weight	Not reported
(Kunczynski and E. Solutions, 2023)	Lettuce (3 cultivars), greenhouse	Descriptive (no predictive model)	Light duration, spectrum	Fixed high-blue nighttime protocol	Phytochemical enhancement	Fixed high-blue LED	None	No CF modeling	N/A	No yield loss; ↑ phytochemicals	Not reported
(Fisher et al., 2001)	Tomato, LED growth chamber	ANOVA-based descriptive	Spectrum type	Static spectrum comparison	Morphology and P_n_ response	Fixed LED spectra	None	No CF consideration	N/A	Spectrum-dependent P_n_ changes	Not reported
(Rahman et al., 2021)	Plant factory (CEA system)	AI cyber-physical model	Multi-sensor (≥4 variables)	AI-based energy optimization	Energy + productivity co-optimization	LED (unspecified spectrum)	IoT-based control	Yes (energy-focused CF reduction)	Not reported	System-level energy savings	~30% energy reduction
(Albright, 2000)	Greenhouse pepper	Life cycle assessment (LCA)	Soil–crop system inputs	Integrated soil-crop management	Carbon footprint reduction	No supplemental LED lighting	None	Explicit LCA-based CF model	N/A	Yield maintained	−15–25% GHG emissions
(Poorter et al., 2022)	Greenhouse cucumber	Regression model	Potassium, irrigation	Fertigation optimization	Resource-use efficiency	Fixed broadband LED	None	No CF model	0.934	+17.8% yield	Not quantified
(Gruda and Jackson, 2023)	Long-day ornamentals	Empirical flowering model	Far-red duration, DLI	Fixed photoperiod protocols	Flowering time reduction	Far-red supplementation	None	No CF modeling	N/A	Faster flowering (days reduced)	Not reported
(California Agricultural Technology Institute)	Solar greenhouse (cold regions)	Thermal–optical simulation	Daylighting, insulation, heat storage	Multi-parameter system optimization	Energy + thermal performance	No spectral LED control	None	Indirect energy-related CF	N/A	Not crop-centered	Energy savings reported

### 3.8 Limitations and future directions

The present study validated the CNN-ELM/MOEA/D/TOPSIS framework within a single greenhouse facility under the specific experimental conditions described in Section 2. No formal benchmarking against alternative multi-objective evolutionary algorithms beyond MOEA/D, or against conventional fixed-schedule lighting systems, was conducted within this study; such comparative evaluation would strengthen claims about the framework’s relative merits and is planned for subsequent research. Furthermore, a formal sensitivity analysis of the MOEA/D hyperparameters — including population size, neighborhood size, differential evolution scale factor, and crossover probability — on the shape and diversity of the resulting Pareto-approximate fronts was not performed in the current work. While the parameter values applied here were selected based on published guidelines for decomposition-based multi-objective optimization in constrained engineering problems, future work should systematically assess how variation in these parameters affects the stability and reproducibility of the TOPSIS-selected operating points across different temperature–humidity conditions. Additionally, the entropy-weighted TOPSIS selection procedure assigns objective importance based solely on the informational content of the current Pareto front; in commercial deployment scenarios where growers have explicit economic preferences between photosynthetic performance and energy cost, incorporating preference-based weighting as an adjustable operator parameter would enhance the practical utility of the decision framework.

Recent advancements in machine learning (ML), intelligent electronics, and functional materials have established a robust technical foundation for optimizing precision agricultural illumination and thermal management in protected tomato cultivation (Dai et al., 2006b; Dai et al., 2007; Tian et al., 2016; Li et al., 2026a; Wu et al., 2026). In ML and intelligent control, data-driven frameworks have achieved notable progress in stochastic system control and robust decision optimization, where stochastic model predictive control has been applied to nonlinear system tracking and distributionally robust self-triggered MPC enhances stability under uncertain scenarios (Navaneethan et al., 2025; Dai et al., 2005; Dai et al., 2006a; Dai et al., 2011; Lv et al., 2025; Li et al., 2026). Cross-modal ML models have advanced visual and data analysis, with spatio-temporal fusion algorithms enabling efficient video deblurring and scene enhancement, and knowledge-driven modulation networks improving semantic feature perception- (Dai et al., 2019; Liu et al., 2020; Zeng et al., 2022; Han et al., 2024; Wei et al., 2025). ML applications have further expanded to smart contract vulnerability detection, decentralized exchange protection, image-text semantic alignment, and real-time task scheduling across complex dynamic environments (Liu et al., 2020; Yang et al., 2022; Shi et al., 2024; Fu et al., 2026; Li et al., 2026). Spatio-temporal vision-language models and advanced scheduling strategies have further verified the strong generalization ability of ML algorithms in complex agricultural and industrial environments (He et al., 2025; Li et al., 2025; Li et al., 2025; Zhu et al., 2025; Ma et al., 2026).

In optoelectronic device technology, wide-bandgap semiconductor advances have driven spectral illumination upgrades, with high-quality ZnO films fabricated via MOCVD using multiple precursors and substrate templates (Tian et al., 2018; Lv et al., 2024; Liu et al., 2025; Zang et al., 2025; Ding et al., 2026). Pulsed laser deposition has further optimized nonpolar ZnO film structural and optical properties for diverse optoelectronic device environments, while novel AlGaN-based far-ultraviolet micro-LEDs and ZnO/AlGaN heterojunction dual-function UV devices provide high-performance spectral regulation components for protected cultivation (Dai et al., 2018; Tian et al., 2018; Li et al., 2026b; Yao et al., 2026; Zong et al., 2026). In functional materials, amphiphilic copolymer viscosifiers enable operation under high-temperature and high-salinity conditions, while shear-thickening mortar composites and hydrophobic polymer-modified materials offer mechanical resistance and structural stability under complex working conditions. Numerical simulations of multi-phase fluid interactions and droplet deformation provide theoretical support for thermal and fluid field regulation in cultivation environments. Cross-disciplinary material–biology studies have revealed regulatory mechanisms of natural bioactive compounds, where perillaldehyde induces apoptosis in Aspergillus flavus via metacaspase pathways and suppresses aflatoxin biosynthesis through FadA-cAMP signaling disruption, pentagalloylglucose and PARP inhibitors synergistically enhance microbial stress sensitivity, and luteolin mitigates oxidative stress through Nrf2 and HIF-1α regulation. Multi-scale entropy analysis, team decision optimization, and EEG-based sensory perception methods offer novel analytical frameworks for evaluating intelligent agricultural system states, while nanobubble–mineral synergistic activation provides new strategies for microenvironmental conditioning in protected cultivation. Further interdisciplinary advances spanning industrial imaging, power electronics, and materials informatics have reinforced the computational and sensing capabilities underpinning intelligent agricultural systems. Specular reflection removal techniques for industrial metal surfaces under fixed lighting configurations have enhanced machine vision reliability in structured environments (Chen et al., 2024), while fault-tolerant multiparallel three-phase converter architectures with adaptive hardware reconfiguration have improved power delivery robustness for agricultural optoelectronic equipment (Zeng et al., 2024), and physics-deep learning hybrid frameworks for materials property prediction offer computationally efficient and interpretable approaches for functional material design (Ren et al., 2025). Machine learning-guided femtosecond laser trepan drilling optimization using high-throughput multi-objective genetic algorithms demonstrates the power of ML in precision manufacturing of agricultural photonic components (Zhang et al., 2022), radar-assisted predictive beamforming for UAV-aided networks via deep learning provides enhanced wireless sensing and communication capabilities relevant to smart farm monitoring infrastructure (Yin et al., 2025), and illuminated fulvic acid-stimulated denitrification and arsenic immobilization in flooded paddy soils via biophotoelectrochemical pathways reveals critical light-driven soil biogeochemical mechanisms applicable to protected cultivation management (Zeng et al., 2024). Novel spatial-temporal learning methods for adaptive video streaming generalization (Zhang et al., 2025), combined phytohormone and nitrogen strategies for enhancing heterotrophic lutein production in Chlorella protothecoides (Li et al., 2025), and physics-informed extreme learning machines for Stefan phase-change problems (Ren et al., 2025) collectively broaden the algorithmic and biological toolkit available for dynamic agricultural environment regulation. Furthermore, lightweight deep-and-wide network architectures for image-based industrial waste gas detection provide efficient environmental monitoring frameworks directly transferable to greenhouse air quality assessment in protected tomato cultivation systems (Gu et al., 2026).

## 4 Conclusion

This study has developed and experimentally validated an integrated intelligent supplemental lighting control framework for greenhouse tomato production that advances the state of the art in three interconnected domains: photosynthesis prediction, multi-objective optimization, and real-time environmental-feedback control. The principal conclusions are as follows.

The CNN-ELM hybrid photosynthesis prediction model, incorporating five environmental inputs including relative humidity, achieved R² = 0.976 and RMSE = 0.712 µmol m^-2^ s^-1^ on an independent held-out validation set of 60 observations, outperforming both the ELM-only baseline (R² = 0.918, RMSE = 1.431 µmol m^-2^ s^-1^) and the standalone CNN (R² = 0.948, RMSE = 1.167 µmol m^-2^ s^-1^) across all five reported performance metrics. The inclusion of relative humidity as a fifth model input produced a measurable and demonstrable improvement over four-variable models — ablation analysis confirmed RMSE degradation from 0.712 to 0.891 µmol m^-2^ s^-1^ upon its removal — establishing its relevance for high-accuracy photosynthesis estimation under the variable humidity conditions typical of winter greenhouse operation. These accuracy figures were achieved within the specific experimental domain of this study: one cultivar (Jinpeng No. 1), one greenhouse facility, temperatures of 18–30 °C, PPFD up to 1800 µmol m^-2^ s^-1^, R:B ratios of 2–8, and ambient CO_2_ near 400 µmol mol^-1^. They should not be interpreted as evidence of transferability across cultivars, CO_2_ enrichment regimes, or seasons with substantially different natural radiation environments; independent external validation under such conditions is recommended before the model is deployed in operational contexts that fall outside this calibrated domain.

MOEA/D-based multi-objective optimization generated well-distributed Pareto-approximate fronts across a 20-point temperature–humidity grid spanning T ∈ {18, 21, 24, 27, 30} °C and H ∈ {40, 55, 70, 85}%, consistently identifying R:B values in the range 6.7–7.2 and temperature-responsive PPFD targets as the optimal spectral-intensity combination for simultaneous P_n_ maximization and LED-associated carbon footprint minimization. The smooth concave curvature of all 20 Pareto fronts confirmed genuine continuous trade-off between the two objectives and the effectiveness of MOEA/D’s Chebyshev decomposition in maintaining solution diversity across the front. Entropy-weighted TOPSIS — which derives objective importance from the informational content of the Pareto solution set without requiring subjective operator preference elicitation — provided a rigorous, reproducible, and automation-compatible procedure for selecting single operational setpoints, yielding entropy weights of w_1_ = 0.52 (P_n_ objective) and w_2_ = 0.48 (C_F objective) that reflect approximately balanced informational contribution of the two objectives across the operating grid.

Bivariate polynomial decision surfaces in T–H space — with LOO-CV R² values of 0.937 (red channel) and 0.921 (blue channel) confirming adequate continuous representation of the discrete optimization results — were deployed on a cloud computational server interfaced with a LoRa wireless sensor network and PWM-controlled LED driver hardware. The LoRa mesh achieved packet delivery ratios of 99.82% (uplink) and 99.90% (downlink) across an 18-node sensor network over a 72-hour evaluation period, and mean PPFD tracking errors of −1.84% (red channel) and −1.62% (blue channel) across the full operating temperature range, both well within the measurement uncertainty of the quantum sensors used for feedback (± 5%). These communication and actuation performance figures confirm operational reliability suitable for the 10-minute control interval employed in this study.

The dual-parameter DP treatment group reduced LED-associated carbon footprint — defined explicitly as the CO_2_-equivalent emissions arising from supplemental lighting electricity consumption only, bounded at the LED subsystem level and calculated using a regional grid emission intensity of 0.5810 kg CO_2_-eq kWh^-1^ — by 38.4% relative to the full-load reference system, while simultaneously improving mean single-leaf P_n_ by 22.6%, total fruit yield by 31.7% (742.6 versus 564.1 g plant^-1^), and total above-ground fresh biomass by 30.5% relative to the unlit AC group over the 110-day production trial. It should be noted that this carbon reduction figure reflects a lighting-specific efficiency gain and does not represent a reduction in total greenhouse operational emissions, which would require a full system-level energy audit encompassing heating, ventilation, irrigation, and auxiliary systems that were outside the defined system boundary of this study. DP consistently exceeded the single-parameter SP group across all productivity metrics — fruit yield by 15.4%, P_n_ at 105 DAT by 8.6%, above-ground biomass by 13.6% — with treatment differences becoming statistically significant at 70 DAT and intensifying through the remainder of the growing season, indicating that short-term trials of fewer than 50 days are unlikely to capture the full productivity benefit of dynamic spectral optimization.

The improvement in the ratio of crop output to lighting energy input demonstrated in this study should be understood as a domain-specific finding rather than evidence of a general decoupling of energy consumption from productivity. Within the operational conditions evaluated — temperatures of 18–30 °C, relative humidity of 40–85%, and the ambient irradiance levels of a winter production season in northern China — the temperature-responsive and humidity-responsive PPFD optimization produced setpoints consistently lower than the full-load maximum while sustaining superior photosynthetic performance relative to both control groups. Whether this energy-yield efficiency advantage extends to substantially different seasons, CO_2_ enrichment conditions, cultivars, or geographic locations with different radiation environments remains an open question that multi-site, multi-season validation studies should address before broad deployment recommendations are made.

## Data Availability

The raw data supporting the conclusions of this article will be made available by the authors, without undue reservation.
