# Supplementary material for: Adaptive spectral–thermal illumination management for protected tomato cultivation: a fused deep learning and pareto-based decision framework
Source: Front Plant Sci. 2026 Jun 29;17:1847258. doi: 10.3389/fpls.2026.1847258 (PMC13357427; doi:10.3389/fpls.2026.1847258)
Supplement: Supplementary file 1 [file Table1.docx]

Adaptive Spectral–Thermal Illumination Management for Protected Tomato Cultivation: A Fused Deep Learning and Pareto-Based Decision Framework

**Kabeer Usman Abdulrazaq^1^*,** **Amuthakkannan Rajakannu^2^**

^1^School of Engineering and Technology, National Forensic Sciences University, Gandhinagar 382007, Gujarat, India:

^2^Department of Mechanical and Industrial Engineering, College of Engineering, National University of Science and Technology, Muscat, Sultanate of Oman.
Email: [amuthakkannan@nu.edu.om](mailto:amuthakkannan@nu.edu.om)

***Corresponding author**: jajasisi1998@gmail.com/[amuthakkannan@nu.edu.om](mailto:amuthakkannan@nu.edu.om)

**Table S1 — LED Array Technical Specifications and Carbon Footprint Model Parameters**

| **Parameter** | **Symbol** | **Value** | **Unit** |
| --- | --- | --- | --- |
| Red channel peak emission wavelength | λ_R | 661 | nm |
| Red channel FWHM | — | 18 | nm |
| Blue channel peak emission wavelength | λ_B | 448 | nm |
| Blue channel FWHM | — | 22 | nm |
| Red channel photon flux efficacy | η_R | 1.42 | µmol·s⁻¹·W⁻¹ |
| Blue channel photon flux efficacy | η_B | 1.28 | µmol·s⁻¹·W⁻¹ |
| Maximum array electrical load | P_max | 420 | W |
| Maximum canopy PPFD at 18 cm | PPFD_max | 2,720 | µmol·m⁻²·s⁻¹ |
| Working distance (lamp face to canopy) | d | 18 | cm |
| PWM resolution | — | 12-bit (4,096 steps) | — |
| Regional grid emission intensity | E_g | 0.623 | kg CO₂·kWh⁻¹ |
| Maximum R:B ratio attainable | R:B_max | 12 | dimensionless |
